# Distribution of genetic diversity reveals colonization patterns and philopatry of the loggerhead sea turtles across geographic scales

**DOI:** 10.1038/s41598-020-74141-6

**Published:** 2020-10-22

**Authors:** Miguel Baltazar-Soares, Juliana D. Klein, Sandra M. Correia, Thomas Reischig, Albert Taxonera, Silvana Monteiro Roque, Leno Dos Passos, Jandira Durão, João Pina Lomba, Herculano Dinis, Sahmorie J.K. Cameron, Victor A. Stiebens, Christophe Eizaguirre

**Affiliations:** 1 School of Biological and Chemical Sciences, Queen Mary University of London, London, E1 4NS UK; 2 MARE-ISPA, Rua Jardim Do Tabaco, 34, 1100-304 Lisboa, Portugal; 3 Molecular Breeding and Biodiversity Research Group, Department of Genetics, Stellenbosch University, Private Bag XI, Stellenbosch, 7602 South Africa; 4 Instituto Do Mar (iMAr), Cova de Inglesa, C.P 132 Mindelo, Ilha do São Vicente Cabo Verde; 5 Turtle Foundation, An der Eiche 7a, 50678 Cologne, Germany; 6 Associação Projeto Biodiversidade, Mercado Municipal 22, Santa Maria, 4111 Ilha do Sal Cabo Verde; 7 Projeto Vitó Porto Novo, Porto Novo, Ilha do Santo Antão Cabo Verde; 8 Foundation Maio Biodiversity, Cidade de Porto Inglês, Ilha do Maio Cabo Verde; 9 Biosfera I, Rua de Moçambique 28, Mindelo, Ilha do São Vicente Cabo Verde; 10 Associação Ambiental Caretta Caretta, Achada Igreja, Pedra Badejo, Santa Cruz, Ilha do Santiago Cabo Verde; 11 Associação Projecto Vitó, Xaguate, São Felipe, Ilha do Fogo Cabo Verde

**Keywords:** Genetics, Population genetics, Evolution, Evolutionary genetics

## Abstract

Understanding the processes that underlie the current distribution of genetic diversity in endangered species is a goal of modern conservation biology. Specifically, the role of colonization and dispersal events throughout a species’ evolutionary history often remains elusive. The loggerhead sea turtle (*Caretta caretta*) faces multiple conservation challenges due to its migratory nature and philopatric behaviour. Here, using 4207 mtDNA sequences, we analysed the colonisation patterns and distribution of genetic diversity within a major ocean basin (the Atlantic), a regional rookery (Cabo Verde Archipelago) and a local island (Island of Boa Vista, Cabo Verde). Data analysis using hypothesis-driven population genetic models suggests the colonization of the Atlantic has occurred in two distinct waves, each corresponding to a major mtDNA lineage. We propose the oldest lineage entered the basin via the isthmus of Panama and sequentially established aggregations in Brazil, Cabo Verde and in the area of USA and Mexico. The second lineage entered the Atlantic via the Cape of Good Hope, establishing colonies in the Mediterranean Sea, and from then on, re-colonized the already existing rookeries of the Atlantic. At the Cabo Verde level, we reveal an asymmetric gene flow maintaining links across island-specific nesting groups, despite significant genetic structure. This structure stems from female philopatric behaviours, which could further be detected by weak but significant differentiation amongst beaches separated by only a few kilometres on the island of Boa Vista. Exploring biogeographic processes at diverse geographic scales improves our understanding of the complex evolutionary history of highly migratory philopatric species. Unveiling the past facilitates the design of conservation programmes targeting the right management scale to maintain a species’ evolutionary potential.

## Introduction

The distribution of species is shaped by environmental variation acting both at macro and micro evolutionary scales^1^. Currently, the distribution of biodiversity reflects species' evolutionary history as well as eco-evolutionary dynamics within and across systems^2^. Because environmental factors (e.g.^3^) and past events of colonization (e.g.^4^) leave genetic signatures, characterizing the distribution of genetic diversity sheds light on the mechanisms underlying the species and populations’ distributions^5,6^. Understanding those mechanisms is crucial for the implementation of management and conservation measures to maintain species’ evolutionary potentials^7,8^. The recent years have seen an increasing number of tools available to perform model-based inferences in evolutionary genetics^9^. Inferences are made over likelihood estimates of a certain set of pre-defined parameters constituting different evolutionary scenarios^10^. Likelihood estimates can be obtained, for example, with exact calculations and usually use coalescent theory and Bayesian statistics to simulate phylogenies with Markov Chain Monte Carlo (MCMC) samplers^11^. Alternatively, likelihood estimates can be obtained with Approximate Bayesian Computation (ABC) methods which emerged as an alternative that derives likelihood estimates through comparisons of summary statistics of simulated datasets with those obtained from observed data^9^. Being less demanding computationally, ABC facilitates hypothesis-testing to explain phylogeographic patterns that would be otherwise challenging to explore through computing exact likelihood estimates. For marine species, model-based inferences have contributed, for example, to understand oceanic divergence of humpback whales or the post glacial distribution of blacknose sharks^12,13^. ABC methods hence are promising to investigate the complex demographic patterns of philopatric, yet highly migratory species.

Philopatry, the tendency of an organism to return to its home area or natal site to reproduce^14,15^, impacts the genetic structure of species, forming groups of individuals of similar matrilineage^16,17^. This evolutionary strategy is common in the aquatic realm [e.g. salmonids^18^, cetaceans^19^, sharks^20^ and turtles^21–23^]. Particularly, sea turtles are capable of homing on a scale of a few kilometres^24^. Upon hatching, neonates enter the ocean, find the major currents, which tend to guide them in actively escaping predator-rich coastal waters^25–27^, and disappear for a period known as the “lost years”^28^. At sexual maturity, turtles return to their natal rookery likely using a combination of geomagnetic and olfactory cues^29, 30^. Given this strong site fidelity, it is not surprizing that some genomic regions demonstrate patterns of local adaptation^16^.

Overall, despite philopatry, sea turtles have colonized various habitats over evolutionary timescales^31–33^. The loggerhead turtle (*Caretta caretta*) in particularly is widely distributed in tropical and temperate regions, with nesting aggregations ranging from South Africa to Virginia (USA), including the world’s largest rookeries located in Florida (USA, ~ 50.000 nests per year) and Masirah Island (Oman; ~ 30.000 nests/year^34–36^. The biogeography of Atlantic loggerheads was hypothetically shaped by geological and climatic events^31^. The first of these events is the closure of the Isthmus of Panama that separated the Atlantic from the Pacific ~ 4.1 M years ago^36,37^. Since then, it has acted as a barrier for species that cannot tolerate freshwater conditions, preventing the movement between the Atlantic and the Pacific Oceans^31,38,39^. The second major biogeographic event refers to the warm water intrusions that have occurred during interglacial periods around the tip of South Africa, originating from the Agulhas Current^40^. These warm inflows may have permitted the movement of loggerhead turtles from the Indian Ocean during interglacial periods. Reversely, when the cold Benguela current predominates in this southern hemisphere region, the lack of low-temperature tolerance may prevent gene flow between both ocean basins^31^.

There are currently two main hypotheses that explain the colonization history of loggerhead rookeries in the Atlantic. On the one hand, it has been proposed that the American rookery, ranging from southern Florida to Northern Carolina, is the oldest rookery in the Atlantic and among the first colonized—a conclusion drawn from the high haplotype and nucleotide diversity detected in this aggregation^41^. On the other hand, Shamblin et al.^36^*.* suggested that the present Brazilian rookeries are the oldest in the Atlantic Ocean. This hypothesis is supported by the basal position of Brazilian mitochondrial DNA (mtDNA) haplotypes in a global haplotype network^36^. Both hypotheses stem from the existence of two divergent mtDNA haplogroups: Haplogroup I—CCA1 and Haplogroup II—CCA2^31,36,38^. Approximately 40 distinct haplotypes identified over 3000 turtles revealed a surprising divergence of up to 34-point mutations separating the two major haplogroups^16,31,36,41^. Despite the suggestion of at least two colonization waves^36^, such scenarios have not been formally tested, separating the history of those haplogroups and their origins within the Atlantic Ocean.

Interestingly, the role of the Eastern Atlantic rookery in a colonization scenario remains to be completely understood^36^. The Eastern Atlantic supports the third largest nesting aggregation of loggerhead turtles in the Archipelago of Cabo Verde^42^. This archipelago is located approximately 600 km off the Western coast of Africa. It consists of 10 volcanic islands with the oldest ~ 20My in the East (Maio) and the youngest aged of ~ 8My in the West^43^. There, turtles lay well over 15.000 nests per year^42^ and this number continues to grow. The majority of nesting events occurs on the island of Boa Vista, Maio, Sal and tends to reduce westwards. The existence of two very divergent lineages suggests that at least two independent colonization events occurred^16,44^. Similarly, the asymmetric distribution of turtle density in the archipelago calls for the investigation of the directionality of gene flow to better understand the pattern of distribution of genetic diversity. Such knowledge will determine the source and sink island-specific nesting groups, facilitating management of this rookery. Noteworthy, here we refer to island-specific nesting groups, the turtles that nest on a given island since they form the local management unit^16^. Nesting density on a smaller geographic scale is also heterogeneous: a beach of 15 km length along the south eastern coast of Boa Vista island supports around > 50% of all nesting activity in Cabo Verde^42^.

In this study, we conducted population genetic analyses on loggerhead turtle rookeries ranging from the large geographic scale of the Atlantic Ocean and Mediterranean Sea, to the regional scale of the Cabo Verde archipelago, and to the local scale of the Island of Boa Vista. We aimed to (1) revisit hypotheses of the Atlantic Ocean colonization to clarify the role of the whole Cabo Verde nesting aggregation in this process, using hypothesis-driven population genetics modelling; (2) determine the impact of philopatry on the distribution of genetic diversity and demographic parameters at various geographical scales from the Cabo Verde archipelago to the island level.

## Results

**Figure 1:**
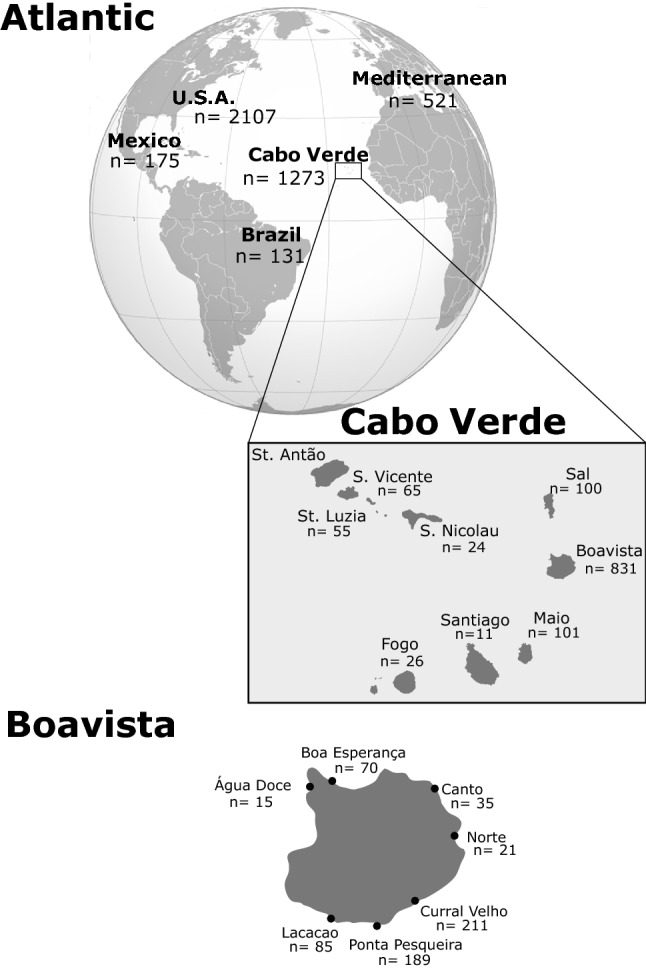
Sample origin across the three geographic regions. Represented are the number of sequences used in this study for each specific geographic scale: Atlantic, regional (Cabo Verde) and local (Island of Boa Vista). These numbers include both the sequences retrieved from the literature as well as those specifically collected for this study.

Firstly, we retrieved 521 sequences of loggerhead turtles from the Mediterranean Sea^22,45–47^, 2107 from the USA, which included the whole South Eastern Coast from South Florida to North Carolina^36,44^, 131 from Brazil^36^, 175 from Mexico^36^ and 392 from Cabo Verde^16,36,44^. In total, 3326 sequences were obtained from the published literature (Fig. 1, supplementary file S1). Hereafter, we refer to those major geographic regions as rookeries. To complement the already existing dataset and improve the resolution at the regional level, field surveys took place in the Cabo Verde archipelago in 2011, 2012 and 2013 during the nesting seasons from June to October. Turtle nesting on nine different islands were sampled: Boa Vista, Fogo, Maio, Sal, Santa Luzia, Santiago, Santo Antão, São Nicolau and São Vicente. Together it resulted in 1273 sequences from Cabo Verde. All new data collected from Cabo Verde are freely available at https://www.qmul.ac.uk/eizaguirrelab/turtlebase/. Because some sequences retrieved from literature, as well as some obtained in this study, had different lengths, all sequences were trimmed to a consensus length of 674 bp from the original ~ 780 bp that compose the long fragments known to loggerhead turtle biologists. This length does encompass the most polymorphic region of the control region^44^ (Fig. 1). In total, this study includes 4207 different sequences (Supplementary file S1).

### On the large-scale rookeries of the Atlantic basin

#### Diversity and population structure

Indices of genetic diversity revealed that the Mexican loggerhead rookery exhibited the highest haplotype diversity (*Hd*_Mexico_ = 0.770), while the USA rookery had the highest value of nucleotide diversity (*π*_USA_ = 0.023). The Mediterranean rookery showed the lowest values of haplotype (*Hd*_Mediterranean_ = 0.348) and nucleotide diversity (*π*_Mediterranean_ = 0.001), suggesting this rookery has been the most recently colonized or is the result of fewer events of colonization. The Cabo Verdean rookery showed one of the highest indices of haplotype diversity (*Hd*_Cabo Verde_ = 0.572) but one of the lowest of nucleotide diversity (*π*_Cabo Verde_ = 0.005, Table 1). This contrasting pattern highlights the importance of placing the Cabo Verde Archipelago in the colonization history of the loggerhead turtles across the Atlantic Ocean.

**Table 1:** Diversity indices of major rookeries of the Atlantic Ocean.

Rookery	n	nHap	Hd	π
Brazil	131	3	0.467	0.001
Cabo Verde	1273	20	0.572	0.005
Mediterranean	521	13	0.348	0.001
Mexico	175	14	0.770	0.015
USA	2107	23	0.555	0.023

*n* number of individual analysed, *nHap* number of haplotypes, *Hd* haplotype diversity, *π* nucleotide diversity.

Not surprisingly given the philopatric nature of loggerhead turtles, pairwise F_ST_ comparisons among global rookeries revealed highly significant differentiation with F_ST_ values ranging from 0.964 (p < 0.001) between the rookeries of Brazil and the Mediterranean Sea to 0.303 (p < 0.001) between the rookeries of Cabo Verde and that of the USA (Fig. S2a, Table S1, Table S2). Exact tests of population differentiation showed similar results (Table 2). On average, the USA rookery showed the lowest, though all highly significant, level of pairwise differentiation among all rookeries. None of the grouping scenarios tested with hierarchical AMOVA revealed significant F_CT_, suggesting that this approach is not informative enough to infer the overall structure among rookeries.

**Table 2:** Exact population differentiation tests among rookeries.

	Mexico	USA	MED	CV
USA	**0.000**			
MED	**0.000**	**0.000**		
CV	**0.000**	**0.000**	**0.000**	
BRA	**0.000**	**0.000**	**0.000**	**0.000**

In bold, significant values for p < 0.01.

#### Ancestry and colonization routes of global rookeries

To infer ancestry and colonization routes across the Atlantic and into the Mediterranean Sea, we used phylogenetic models constructed in BEAST v.1.8^48^, where ancestry and monophyly were enforced sequentially for all rookeries. The comparison of marginal likelihoods of phylogenetic models with fixed ancestry suggested the USA rookery to be the oldest among all the ones analysed (Table S3). Ranking AICM values across models strongly suggest the Mediterranean rookery to be youngest. On the other hand, comparisons of Migrate-n^49^ models based on Bayes factors revealed a model with an ancestral Mexico rookery from which turtles colonized all others rookeries. This best model also suggests a young Mediterranean rookery and a central Cabo Verde rookery acting as a stepping stone towards both sides of the Atlantic (best fit model M4, probability of 0.63, Fig. S1, Table S4). These results appear contradictory and explain the previously uncertain colonization history of this Atlantic for this species.

**Figure 2:**
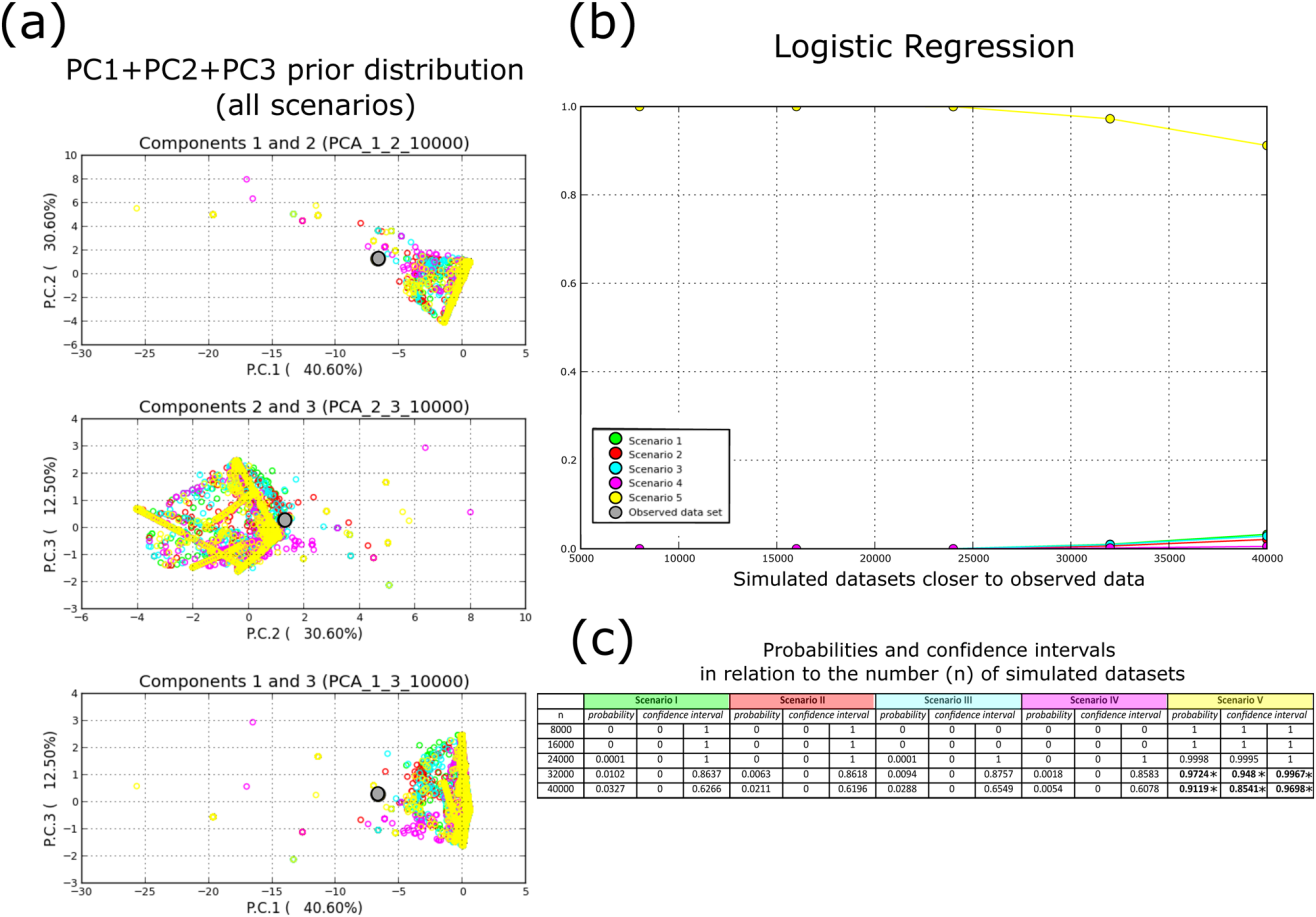
Results of hypothesis-testing generated scenarios with Approximate Bayesian Computation. In (**a**), prior distributions of simulated datasets for all the 5 scenarios in relation to the observed dataset (figure produced by the software). In (**b**), is shown the multinomial logistic regression used to infer the best-fit model considering a gradual increase of simulated datasets, *n,* ranked by proximity to the observed dataset. Specifically, the x-axis denotes the number of simulated datasets closer to the observed dataset, *n* = {8000, 16,000, 24,000, 32,000, 40,000}; the y-axis shows the logit regression R^2^ as a function of *n*. In (**c**) is given the evolution of R^2^ ´s confidence intervals for the *n* in (**b**). The * denotes non-overlapping confidence intervals among scenarios for the same *n.* The figure was partially produced with DIYABC v2.1.0.

**Figure 3:**
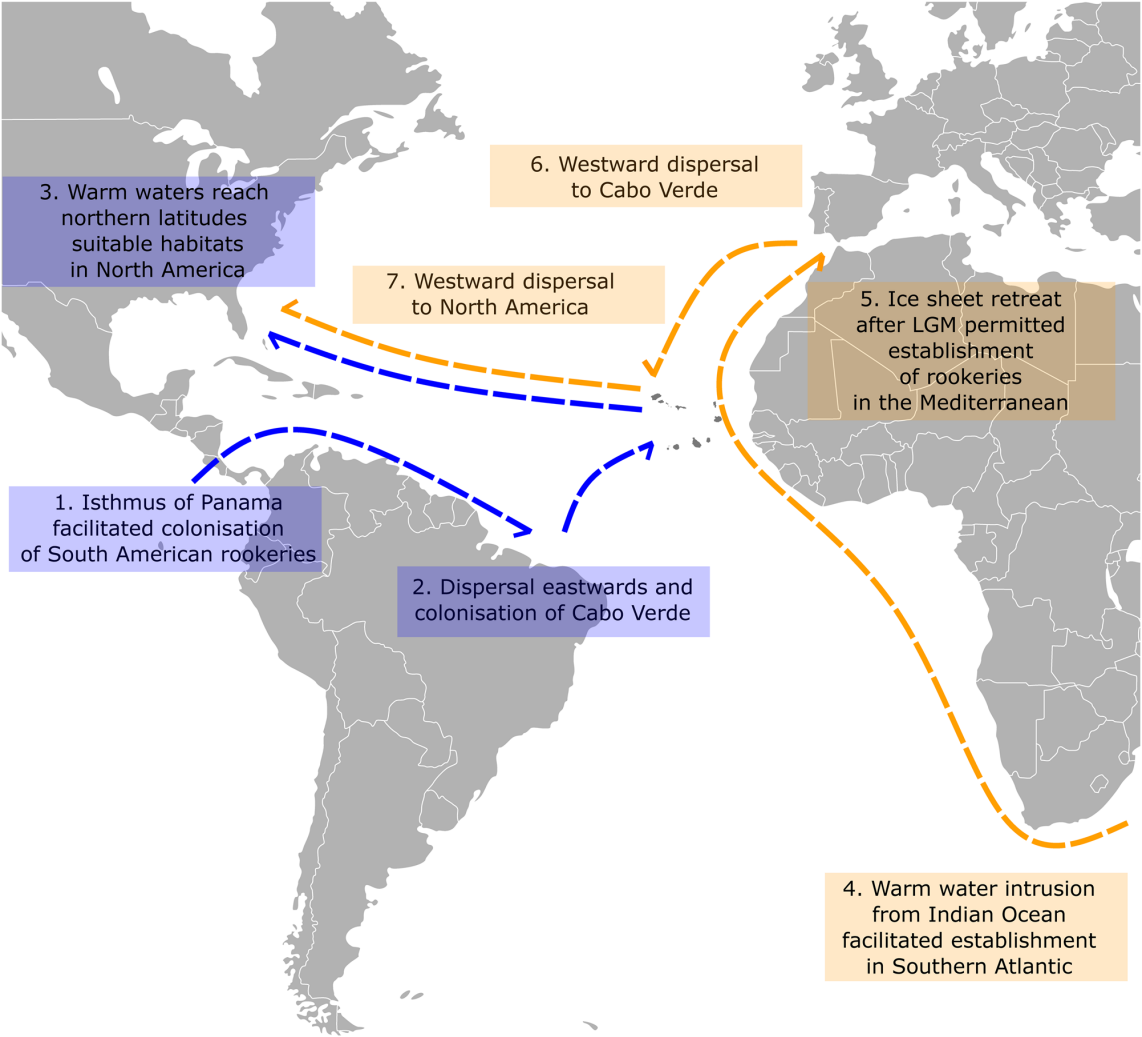
Reconciling the hypotheses of the colononization of the Atlantic Ocean. Visual representation of the colonisation and dispersal model of the haplogroups I-CCA1 and II-CCA2, supporting two colonisation waves, one via the Isthmus of Panama and the other, later, via South Africa. The figure also highlights the role of the central Atlantic Cabo Verdean rookery as a stepping stone for each lineage across the Atlantic Ocean.

Hence, we complemented this set of models with Approximate Bayesian Computations splitting the origin of the two haplogroups (Fig. S3, Fig. S4). This approach suggested a different model to be the most likely (Fig. 2, Fig. S5). Indeed, in our scenario S5, Brazil is likely the most ancient rookery in the Atlantic, founded by an ancestral haplogroup I population that later colonized the Cabo Verdean rookery before a return towards the area of USA/Mexico. This S5 scenario further implies that the Mediterranean Sea was the last rookery to be founded but only by haplogroup II, whose individuals dispersed then to USA/Mexico and from there to Cabo Verde archipelago (Fig. 3). Model inferences considering a lineage split prior to colonization and dispersal had never been attempted, and here we show that doing it allows to explore the likelihood of previously theorized colonization scenarios^36,41^.

### Regional level of the Cabo Verde archipelago

With Cabo Verde appearing as a central rookery acting as a stepping stone along the colonization pathway of the Atlantic basin, we investigated the demographics, diversity and population structure of this rookery. For the following analyses, we used 1273 mtDNA sequences of nesting female turtles. Twenty-two different haplotypes were detected, among them three haplotypes (UH5,UH9 and UH13, Supplementary File S1) that were found in previous study (Stiebens et al., 2013b) but not yet described in Genbank or the Archie Carr centre for Sea Turtle Research.

#### Demographic history of the archipelago

We investigated the variation in various demographic indices for each island within the rookery. Results revealed two distinct patterns. The first one describes a possible population expansion in most of the northern set of islands-specific nesting groups of Sal, Santo Antão, São Nicolau, and Boa Vista. For those nesting groups, as well as that in Fogo, Tajima’s were negative but non-significant, and both SSD and raggedness indices showed to not be significant (Table 3). However, São Nicolau exhibited a negative and significant Tajima’s D. The other island-specific nesting groups rather experienced a constant population size or a decline as seen in São Vicente, Santiago, Santa Luzia and Maio islands (Table 3). Amongst those, only São Vicente showed significant and positive Tajima´s D and Fu´s Fs.

**Table 3:** Diversity indices for the island-specific nesting groups of Cabo Verde.

Island	n	nHap	Hd	π	Tajima's D	SSD	r	Fu's Fs
Boa Vista (BV)	831	16	0.564	0.004	− 1.486	0.067	0.269	1.593
Fogo (FG)	26	4	0.640	0.001	− 0.390	0.004	0.089	− 2.187
Maio (MAI)	101	8	0.566	0.001	0.074	0.061	0.255	− 1.994
Sal (Sl)	100	8	0.582	0.007	− 0.964	0.027	0.118	**5.192**
São Nicolau (SN)	24	6	0.681	0.006	− **2.222**	0.009	0.041	2.119
São Vicente (SV)	65	10	0.662	0.020	**2.189**	0.112	0.171	**9.349**
Santa Luzia (SU)	55	3	0.560	0.002	0.879	0.039	0.147	0.562
Santiago (ST)	11	3	0.564	0.001	1.176	0.053	0.251	0.477
Santo Antão (SA)	60	5	0.438	0.001	− 0.760	0.000	0.113	− 1.537

*n* number of individual analysed, *nHap* number of haplotypes, *Hd* haplotype diversity, *π* nucleotide diversity, *SSD* sum of squared differences from mismatch distribution, *r* raggedness index and Fu's Fs. In bold, significant values for p < 0.05.

Reconstructing the effective population size estimates from Bayesian skyline plots revealed that São Vicente, Boa Vista, São Nicolau and Sal showed a recent decline in effective population size, though Boa Vista and São Vicente engaged in a possible recovery close to present time (Fig. S6). An ongoing demographic recovery is in line with negative Tajima D values for Boa Vista. However, the moment-based estimates (Tajima´s D and Fu´s Fs) for São Vicente do indicate a decline, suggesting that the bottleneck that had occurred prior to the ongoing expansion had a pronounced impact on the groups´ genetic diversity. Nesting groups on the islands of Fogo, Maio, Santa Luzia, Santiago and Santo Antão instead show a relatively stable effective population size, and only for the nesting groups in Maio and Santa Luzia did Tajima’s D agree with Bayesian skyline plots.

#### Diversity and structure of each island

Investigating the different haplotypes, we found that the most frequent were CCA1.3 (n = 766) and CCA17.1 (n = 294), both belonging to the oldest Haplogroup I—CCA1 lineage. As expected, haplotype and nucleotide diversities showed less variation at the regional level than at the global level, however they remained high and diverse (Table 3). Indices of genetic diversity split for islands showed that turtles sampled in São Nicolau harboured the highest haplotype diversity (*Hd*_SaoNicolau_ = 0.681) while those from São Vicente showed the highest nucleotide diversity (*π*_SaoVicente_ = 0.020). Both islands belong to the northern area of the archipelago. The lowest values of haplotype diversity were observed in turtles from Santo Antão (*Hd*_SantoAntao_ = 0.438) and the lowest nucleotide diversities were detected in turtles from four islands: Santo Antão, Santiago, Fogo and Maio (*π* = 0.001, Table 3). Noteworthy, the last three islands are adjacent in the Southern part of the archipelago.

Pairwise F_ST_ comparisons among island-specific nesting groups resulted in twelve statistically significant comparisons after Benjamini-Yekutieli false discovery rate (FDR) correction for multiple comparisons (Fig. S2b). The genetic composition of São Vicente Island produced the highest F_ST_ values among island pairs, with statistically significant pairwise F_ST_ ranging from 0.174 with Fogo (p = 0.005) to 0.294 with Maio (p < 0.001) (Table S5). Exact tests of population differentiation showed seventeen significant results, mostly consistent with pairwise F_ST,_ particularly those involving the islands of São Vicente, Fogo and Sal (Table 4). F_ST_ did not correlate with geographic distance (Mantel test: r = -0.138 p = 0.820)_,_ suggesting an absence of isolation by distance.

**Table 4:** Exact population differentiation tests among islands of the Cabo Verde Archipelago.

	Boa Vista	Sal	São Vicente	São Nicolau	Fogo	Maio	Santa Luzia	Santo Antão
Sal	0.140							
São Vicente	0.000	**0.000**						
São Nicolau	0.039	0.195	0.076					
Fogo	**0.001**	**0.007**	**0.000**	0.098				
Maio	0.335	0.020	**0.000**	**0.006**	**0.003**			
Santa Luzia	0.151	0.029	**0.000**	**0.006**	**0.000**	0.100		
Santo Antão	**0.000**	**0.000**	**0.000**	**0.009**	**0.000**	**0.000**	**0.000**	
Santiago	0.933	0.764	0.237	0.623	0.326	0.777	0.747	0.015

In bold, significant values for p < 0.05.

**Figure 4:**
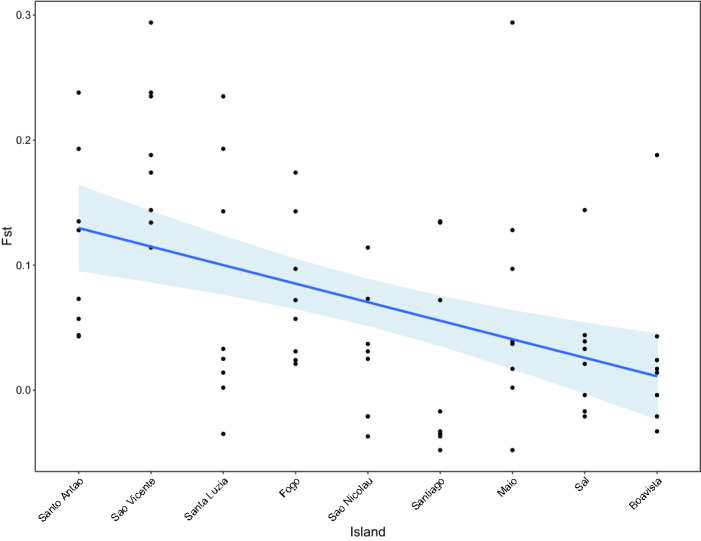
Distribution of average pairwise F_ST_ among the island-specific nesting groups of the archipelago. Linear regression of average pairwise F_ST_ per island along the East to West axis of the archipelago. A significant increase in average pairwise F_ST_ can be observed from East to West (R^2^ = 0.275, p < 0.001).

Interestingly, the average pairwise F_ST_ significantly varied across island-specific nesting groups (ANOVA: F = 4.367, p < 0.001), increasing in a linear manner from East to West (R^2^ = 0.275, p < 0.001, Fig. 4). To further describe this East–West cline, we estimated the number of migrants amongst islands and the direction of gene flow using the migration estimates obtained with Migrate-n. We found a significant interaction between the geographic distance among islands and the direction of gene flow: the average number of migrants decreased eastwards (t_direction West_ = -1.281, p = 0.047) and increased westwards (t_direction West_ = 0.719, p = 0.015). This result demonstrates that the islands with higher turtle abundance in the east, such as Boa Vista, act as sources of migrants towards the other islands westwards (Fig. S7) and as such deserve specific conservation measures.

### Genetic diversity and structure within the island of Boa Vista

Specifically, among the island-specific nesting groups, the island of Boa Vista supported the largest nesting group in the archipelago. We used the largest dataset collected from specific beaches in the island (N = 626) (Fig. 1) to investigate the genetic diversity and population structure at the local scale. We found that the beach of *Agua Doce* showed the highest haplotype and nucleotide diversity (*Hd*_AguaDoce_ = 0.752, *π*_AguaDoce_ = 0.009), while Boa Esperança exhibited the lowest values in both indices (*Hd*_BoaEsperança_ = 0.331, *π*_BoaEsperança_ = 0.001). Interestingly, those beaches are adjacent to each other. Furthermore, haplotype diversity observed at the local scale was comparable to that of the regional level but reduced of about half for the nucleotide diversity (Table 5).

**Table 5:** Diversity indices across beaches in Boa Vista island.

Beach	n	nHap	Hd	π	Tajima's D	SSD	r
Agua Doce (AD)	15	5	0.752	0.009	− **2.005**	0.024	0.065
Boa Esperança (BE)	70	6	0.331	0.001	− 0.871	0.059	0.465
Canto (CA)	35	5	0.51	0.004	− **2.374**	0.118	0.474
Curral Velho (CuVe)	211	9	0.571	0.003	− **2.043**	0.088	0.333
Lacacão (La)	85	10	0.612	0.003	− **2.285**	0.030	0.121
Norte (No)	21	6	0.695	0.006	− **2.209**	0.043	0.145
Ponta Pesqueira (PP)	189	7	0.509	0.005	− 1.179	0.090	0.384

*n* number of individual analysed, *nHap* number of haplotypes, *Hd* haplotype diversity, *π* nucleotide diversity, *SSD* sum of squared differences from mismatch distribution, *r* raggedness index. In bold, significant values for p < 0.05.

Surprisingly, some pairwise comparisons showed to be significant after FDR correction, even at this local scale. All significant tests included the beach of Boa Esperança (Fig. S2c): Boa Esperança and Agua Doce (F_ST_ = 0.220, p = 0.000), Boa Esperança and Curral Velho (F_ST_ = 0.062, p = 0.00), Boa Esperança and Lacacão (F_ST_ = 0.051, p = 0.002) and Boa Esperança and Norte (F_ST_ = 0.114, p = 0.000, Table S6). Given that we sampled 70 turtles from Boa Esperança, those significant results are unlikely to stem from a small sample size. Exact tests of population differentiation showed significant results on pairwise comparisons involving the locations of Ponta Pesqueira and Boa Esperança (Table 6), in agreement with pairwise F_ST_.

**Table 6:** Exact population differentiation tests among the beaches of Boa Vista island.

	Agua Doce	Canto	Curral Velho	Lacacão	Norte	Ponta Pesqueira
Canto	0.028					
Curral Velho	0.035	0.169				
Lacacão	0.283	0.350	0.286			
Norte	0.763	0.178	0.023	0.140		
Ponta Pesqueira	**0.002**	0.085	0.023	**0.001**	0.011	
Boa Esperan-a	**0.000**	0.117	**0.000**	**0.001**	**0.000**	**0.000**

In bold, significant values for p < 0.05.

Turtle sampled on the different beaches of Boa Vista showed a pattern of population expansion as suggested by negative Tajima’s D and non-significant SSD and raggedness index. Those results are consistent with the apparent scenario of population expansion detected at the entire island-specific nesting group (Table 5). Bayesian skyline plots, however, revealed that turtles nesting on Norte, Canto and Agua Doce beaches might have experienced a short population decline, while turtles nesting on Boa Esperanca beach experienced a short expansion, when groups of turtles nesting on the other beaches showed no change in population size (Fig. S8).

## Discussion

To effectively manage endangered species, it is crucial to understand processes that result in the observed distribution of genetic diversity. In this study, we evaluated the distribution of genetic diversity of loggerhead sea turtle from the Atlantic Ocean, known for their natal philopatric behaviours. While philopatry reduces gene flow among populations, the loggerhead turtle has successfully colonized this entire Ocean but the routes underlying this colonization still remain elusive. Here, we show support for the Brazilian rookery to be the most ancient rookery in the Atlantic, further suggesting that the colonization of the entire Atlantic Ocean basin occurred via two waves: one from the Pacific and other from the Indian ocean. Each colonization event corresponds to that of a major haplogroup, which are now distributed across most of the Atlantic rookeries. Furthermore, we show that the Cabo Verde rookery has played a key role in the colonization process, acting as a stepping stone facilitating the establishment of other rookeries on either side of the Atlantic. Focusing particularly on Cabo Verde, we also show that the island supporting the largest nesting density is not necessarily that with the highest genetic diversity indices. This is likely the result of an asymmetric functioning of the different island-specific nesting groups of the archipelago, with the Eastern nesting groups acting as sources and the edge of the distribution, in the West, mostly acting as a sinks. The eastern islands are those where turtle densities are the highest and we find that because of strong philopatry, ~ 10 kms accuracy, exchanges are partly limited.

The existence of two highly divergent mitochondrial haplogroups that shape the phylogeographic patterns of the ocean basins underlies the loggerhead’s global biogeography^33,38^. The Atlantic being the youngest ocean basin, the unequal distribution of mtDNA haplogroups among rookeries suggests different colonization waves, as those lineages stem from the Indo-Pacific area^36^. Therefore, to understand the colonization and posterior formation of the Atlantic rookeries, we considered those haplogroups as independent ancestral populations rather than forming one joint source of colonization. Modelling the temporal succession of haplogroup dispersal into and within the Atlantic Ocean with Approximate Bayesian Computations reconciles current colonization hypotheses^36,41^ under a single scenario. More specifically, the most parsimonious succession of events suggests that the Brazilian rookery was colonized first by individuals carrying haplotypes of the major haplogroup I—probably a leakage from the Pacific into the Atlantic while the Central American Seaway was still present. This was followed by the colonization of the Cabo Verdean archipelago and then the North and Central American rookeries of USA and Mexico. The first stage of the Atlantic Ocean colonization is in line with Shamblin et al. (2014)’s hypothesis, as the Brazilian rookery is the oldest in the Atlantic and is composed only by the turtles carrying haplotypes of the haplogroup I. The colonization from Cabo Verde to north America can be explained by the warm saline waters that were gradually introduced to northern latitudes as the Central American Seaway shallowed until its total closure^50^. As loggerhead turtles do not tolerate cold water and require sand temperatures of at least 25° for successful nest incubation^31^, North American habitats might not have been suitable for nesting until after the closure of the isthmus of Panama.

On a more recent timescale, individuals carrying haplotypes of the haplogroup II colonized the Mediterranean Sea. Likely after the last glaciations, migration occurred to Cabo Verde, where haplogroup II individuals established on already formed colonies, further proceeding to populate USA/Mexico. The pathway here presented thus supports Shamblin’s et al. hypothesis, specifically, the retention in the Indian ocean and the subsequent entry into the Atlantic Ocean via South Africa^36^. The major haplogroup II pathway favoured by the scenario modelling supports the nearly consensual hypothesis that the Mediterranean Sea supports the youngest rookeries with connection to the Atlantic Ocean^45^.

Our biogeographic reconstruction of colonization pathways that assumes two distinct haplogroups as distinct ancestral populations clarifies key elements of the genetic distribution in loggerhead turtles in the Atlantic Ocean. On the one hand, our colonization pathway rejects Central and North American rookeries as the oldest in the Atlantic. However, our summary statistics of diversity and F_ST_, combined with testing colonization hypotheses suggests USA and/or Mexico as the oldest rookeries. The apparent paradox can be explained by the physical convergence of the two divergent lineages in two waves of colonization in this region of the Atlantic Ocean. The admixture of lineages from multiple genetic backgrounds is well known to increase diversity at sink populations^47^. Thus, by being the last rookery to receive individuals carrying the highly diverse haplogroup I, USA/Mexico hold a signature that could be interpreted as that of an ancestral rookery, as observed for loggerhead turtles by Reis et al. (2010). Overall, the Atlantic can be understood as a secondary contact zone of major haplogroups that have accumulated divergence in allopatry. While we cannot exclude demographic effects that could have erased the presence of genetic variants at a local scale, one can parsimoniously assume that once admixed rookeries became established, those demographic effects would affect haplogroups differentiation equally.

Regionally, the Cabo Verdean rookery shows both high haplotype and nucleotide diversities with the presence of both mtDNA haplogroups. Particularly, turtles nesting on the island of São Vicente exhibit a nucleotide diversity at least 3 times higher than that observed in other island-specific nesting groups of the archipelago. This is mainly linked to the presence of the most divergent haplotype CC.2 being frequent on this island. The presence of this haplotype can be traced mostly to one specific beach called Lazareto on the North-West of the island representing ~ 70% of the individuals. The presence of this Indo-Pacific haplotype also present in the Mediterranean Sea is interpreted as evidence for a second wave of colonization^16^. This hypothesis is further reinforced by our scenario simulations of the Atlantic Ocean colonization.

From a population structure point of view, Cabo Verde was considered a single nesting population^44^ and management unit^36^, while early signs of differentiations have been detected^16^. The analyses of the more extensive dataset in this study confirms the highly significant genetic differentiation congruent with both F_ST_ and exact tests. This structure arises clearly from the island of São Vicente and the frequent divergent haplotype but not only. Indeed, a clear geographic pattern exists where the island-specific nesting groups from the western range of the archipelago show increased average differentiation compared to those of the eastern range. Noteworthy, signatures on mtDNA stem from female-mediated gene flow. Because bi-parentally inherited markers show an increase gene flow eastwards linked to opportunistic mating from Western males encountering more females on the East^16^, our results suggest that the densely-populated easternmost islands of Sal, Boa Vista and Maio are extremely important in maintaining the functioning of the rookery, acting as evolutionary source populations, and therefore requiring specific management. Here, we also reveal another geographic pattern, where gene flow of nesting females has propagated from the centre of the distribution to the edge. This pattern might be the ancient signature of colonization within the Archipelago: after the evolution of site fidelity to the firstly colonized islands, populations were established in all other islands through sporadic nesting events from the long-distance migrants, as it has recently been described to occur in the Mediterranean Sea^51^. Altogether, the observed genetic structure is likely the result of a strong female philopatric behaviour as often observed in loggerhead turtles^52, 53^.

From a demographic perspective, estimates of effective population sizes suggest that after a small decline, the most abundant island-specific nesting groups show expansion. This is particularly the case for an East–West corridor entailing Boa Vista to São Vicente islands. While dating the decline is complex, the Cabo Verde rookery may hold the signature of intense poaching which has significantly impacted the census population size^54^. This signature does not necessarily represent the recent poaching peaks of the 1990s and 2000s. Instead, it may be the footprint of poaching activity, known since the human occupation of the archipelago in the fifteenth century^55^.

At the local scale, our results show that turtles nesting on one beach of the island of Boa Vista, Boa Esperança, harbour the lowest nucleotide diversity. This beach is situated about 12kms away from Ponta Ervatao & Ponta Cosme, the beaches with the highest nesting density in Cabo Verde and in the eastern Atlantic^42^. Reduced genetic diversity is likely the result of genetic isolation as demonstrated by the consistent significant differentiation of turtles nesting on Boa Esperança with turtles nesting on the surrounded beaches. Given that the sample size from this beach is ~ 70, this significant differentiation is unlikely to arise from random fluctuation linked to a small sample size. The parsimonious explanation is a high accuracy of philopatric behaviour on Boa Vista which can be detected away from the centre of the nesting activity, on the other side of the island, as the result of leakage of nesting behaviours.

## Conclusion and future directions for turtle conservation

Our results offer a fresh perspective on the biogeography of the loggerhead sea turtle. Understanding the Atlantic Ocean as a secondary contact zone for two highly divergent lineages showed that current hypotheses can be merged into a single colonization and dispersal scenario. The expansion pattern exposed here allows to investigate major climatic and geological changes that shaped the pathways of colonization—such as the impact of the gradual closure of the isthmus of Panama in opening suitable nesting habitats in Northern latitudes. Our work also suggests that interpreting ancestry from genetic diversity estimates should be done cautiously. As we have shown here, the presence of divergent lineages does not necessarily imply ancestry. Hypothesis testing with population genetic modelling may help to clarify conflicting views on global biogeography questions. Despite extensive migration, significant degrees of population differentiation spanned all the analysed geographic levels. Therefore, we have demonstrated that turtles are capable of extreme site fidelity and of forming independent nesting groups at highly localized geographic scales. Lastly, the role of Cabo Verde as the stepping stone connecting major rookeries on each side of the Atlantic supports the critical need to maintain protecting and monitoring loggerhead census sizes across this archipelago^16,29^. In addition, and due to the highly philopatric behaviour that has been consistently reported in this species, and largely reinforced with this work, conservation-driven actions should be enhanced to preserve the genetic biodiversity at the island-level.

## Methods

### Global screen for mitochondrial sequences

Sequences of the mitochondrial control region were obtained both from previously published studies as well as by our own data collection for Cabo Verde. The objective was to obtain a robust representation of the rookeries of loggerhead turtles in the Atlantic and the Mediterranean Sea. We retrieved 521 sequences from rookeries in the Mediterranean Sea^22,45–47^, 2107 from the USA, which included the whole South Eastern Coast from South Florida to North Carolina^36,44^, 131 from Brazil^36^, 175 from Mexico^36^ and 392 others from published studies in Cabo Verde^16,36,44^. In total, 3326 sequences were obtained from published literature, (Fig. 1, Supplementary file S1). For simplicity, these regions are referred to as “rookeries”, though we acknowledge they encompass multiple nesting aggregations.

### Field sampling, DNA extraction and sequencing of mitochondrial control region from Cabo Verde

To complement the already existing dataset and improve the resolution at the regional level, field surveys took place in the Cabo Verde archipelago in 2011, 2012 and 2013 during the nesting seasons from June to October. Turtles nesting on nine different islands were sampled: Boa Vista, Fogo, Maio, Sal, Santa Luzia, Santiago, Santo Antão, São Nicolau and São Vicente. On the island of Boa Vista, where the turtle density is the highest^42^, we sampled on eight different beaches in order to investigate the genetic structure at a local scale. In total, we collected 881 samples from nesting loggerhead females. Samples consisted in removing 3 mm piece of non-keratinized tissue from the right front flipper using a single-use disposable scalpel. Samples were immediately stored in ethanol. Turtles were tagged with metal tags and/or pit tags directly after egg deposition to track nesting behaviours and to avoid multiple sampling^16,56^. In the laboratory, each sample was washed in distilled water for about 20 s and cut into smaller pieces. DNA was extracted using the DNeasy 96 Blood & Tissue Kit (QIAGEN, Hilden, Germany). Elution was conducted in twice 75 μl of AE Buffer. All other steps followed the manufacturer’s protocol.

The long fragment (~ 780 bp) of control region of the mitochondrial DNA was amplified using the Primers LCM15382 (5′-GCTTAACCCTAAAGCATTGG-3′) and H950 (5′-GTCTCGGATTTAGGGGTTTG-3′)^44^. A 10 μl PCR reaction consisted of 1 μl 10 × Buffer, 1 μl dNTP’s (10 mM), 0.3 μl MgCl_2_ (5 nM), 3.6 μl HPLC water, 0.1 μl Taq Polymerase (Invitek), 1 μl of each primer (5 pmol/μl) and 1 μl template DNA (~ 20 μg). The reactions were carried out under the following thermo-cycling conditions: An initial denaturation step of 95 °C for 2 min, followed by a second cycle that was repeated 40 times with denaturation at 95 °C for 30 s, annealing at 55 °C for 30 s and elongation at 72 °C for 1 min. A final elongation step of 7 min at 72 °C was carried out.

PCR products were cleaned with ExoSAP-IT following the manufacturer’s protocol. Cycle sequencing reactions were performed with Big Dye Terminator v3.1 Cycle Sequencing Kit (Applied Biosystems, Darmstadt, Germany). Sequences were obtained from the forward direction (primer LCM15382). Where insufficient fragment lengths were retrieved, sequences from the reverse direction were also obtained and sequences were concatenated into contigs. Sequencing was performed with an ABI 3730 Genetic Analyzer (Applied Biosystems, Darmstadt, Germany). Sequences were assembled in Codon Code Aligner v5.0 (CodonCode Corporation, Dedham, Massachusetts) and ambiguities were corrected by hand investigating carefully the electropherograms for heteroplasmy for instance. All the amplified mitochondrial sequences were classified accordingly to the standardized nomenclature of the Archie Carr Centre for Sea Turtle Research (https://accstr.ufl.edu). The entire data set was aligned in Muscle v8.3.1^57^. All unique sequences can be found in supplementary file S1.

### On the large-scale rookeries of the Atlantic basin

#### Genetic diversity and population structure

Haplotype (Hap) and nucleotide diversity (π) indices were computed in Arlequin v3.5.1.3^58^ for each of the major rookeries in the Atlantic and Mediterranean Sea. Relationships among haplotypes were investigated in a neighbour-joining network using Network v4.6.1.3 (https://www.fluxus-engineering.com), and visualized with PopArt (https://popart.otago.ac.nz). Population structure amongst those major rookeries was investigated using F_ST_ estimates in Arlequin v3.5.1.3 (10.000 permutations). Because the role of Cabo Verde rookery is still yet to be clarified in the broader context of rookeries’ structure, we performed a hierarchic Analysis of Molecular Variance (AMOVA) considering different grouping scenarios, i.e. grouping Cabo Verde with all rookeries separately. The considered rookeries were Brazil, Mediterranean Sea, Mexico and USA. Most likely grouping was identified based on the F_CT_ statistics.

#### Testing the ancestry of Atlantic and Mediterranean rookeries

Rookeries’ ancestry and colonization routes within the Atlantic basin and the Mediterranean Sea were explored by comparing the likelihood ratios of model-based inferences. Phylogenetic models were alternatively built with either the Mediterranean, Mexican, Cabo Verdean, USA and the Brazilian rookery as fixed at the root of the tree^31,36,41^. The initial tree root height was set to initial split between the genus *Caretta* and the genus *Lepidochelys*, 4.09 million years before present^37^. Monophyly was enforced in all rookeries in order to constrain tree topology during the course of MCMC sampling. These analyses were performed in BEAST v.1.8^48^ and based on haplotype sequences without considering their frequencies. The substitution model was set to HKY as it was found to be the best-fit model of nucleotide substitution chosen through Akaike Information Criteria (AICc) in Mega v6.06^59^ and the mutation rate was set to 3.24*10^–3^ substitutions/site/million year, as estimated for marine turtles^37^. The tree prior was set to coalescent and constant size. The MCMC chain length was set to 10^8^ steps. Convergence was inspected in Tracer v1.6^60^, and models were compared by applying the AICM criteria (1000 bootstraps)^61^.

#### Testing colonization scenarios within the Atlantic Basin

To complement the Bayesian phylogenies intended to infer the most likely ancestral rookery, we built possible colonization models in order to (1) strengthen phylogenetic conclusions and (2) infer colonization routes within the Atlantic basin. Colonization hypotheses were tested with models that weight the roles of migration, i.e. gene flow, and mutations as sources of genetic novelty within a population. Colonization models were built and compared in the software Migrate-n v.3.6 through Bayesian inference^49^. Models consisted of different scenarios of rookeries serving as source of migrants, from which only emigration was allowed to occur. In total, we explored 12 possible colonization hypotheses (Fig. S1). Due to the extensive computation power required, we used only unique haplotype data in our preliminary screen. For these models, prior distribution for *gene flow* (M) and *effective population size* (θ) were set as uniform with upper and lower boundaries explored by preliminary tests (θ = 0–20, M = 0–200). Thermodynamic integration with 4 chains with different temperatures (1.0, 1.5, 3.0 and 1,000,000.0) was performed in order to improve the search for parameter space and allow comparisons of models with Bayes factor. A total of 5 × 10^5^ steps were recorded in each chain after a burn-in of 10^4^ states. Three independent replicates were performed for each scenario within each run, in order to ensure convergence. A total of 1.5 × 10^8^ parameters values were visited. Marginal likelihoods were used for model comparisons with Bayes Factor^62^.

We further explored colonization hypothesis with approximate Bayesian computation implemented in the software DIYABC v2.1.0^63^. DIYABC allows the generation of simulated datasets and selection of those closest to the true dataset, and the estimate posterior distribution of specific statistics. The objective was to test the possibility that the two major mtDNA Haplogroups (Haplogroup I—CCA1 and Haplogroup II—CCA2) have distinct evolutionary and colonization histories after the split from a common ancestor.

The genetic composition of contemporary Atlantic rookeries would therefore reflect several instances of secondary contact between proto-populations composed by individuals carrying haplotypes belonging to Haplogroup I—CCA1 and Haplogroup II—CCA2. DIYABC scenarios were built to test both migrate-n the 3 highest rank models and two others that could not be tested with migrate-n due to increased structure complexity.

In total, we constructed and compared 5 scenarios (Fig. S5). Reference tables were built with 4.000.000 simulated datasets. Runs were optimized to search for the summary statistics with the least distance between simulated and observed datasets (Fig. S9). Hence, we used all F_ST_ pairwise comparisons among rookeries from the observed dataset to situate our data with the simulated parameter spaces of the 5 scenarios. Priors were defined as following: uniform population sizes min = 100, max = 500; uniform branching times (in generations) calibrated for the Last Glacial Maximum (LGM) at t1, considering turtle generation time of 50 years—estimated for *Chelonia midas* to be of 46 years^64^. Thus, the t1 priors, in turtle generations, of min = 80, max = 100 place the foundation of the Mediterranean rookery approximately 4000 to 5000 years ago, safely outside the estimates of LGM for the Northern Hemisphere, which is estimated to have taken place approximately 20.000ya^65,66^. The priors for the remaining t were as following: t2 min = 2000, max = 3999; t3 min = 4000, max = 5999; t4 min = 6000, max = 8999 and t5 min = 9000, max = 10,000. Mutation rate was also allowed to vary uniformly between 10^–3^ and 10^–7^, with substitution model Kimura-2.

### Regional level of the Cabo Verde archipelago

#### Genetic diversity and structure within the Cabo Verde archipelago

Arlequin v3.5.1.3 was used to calculate the nucleotide and haplotype diversity for each island-specific nesting group, to compute Wright’s fixation index (F_ST_) and perform exact tests for estimation of population differentiation (10.000 permutations). With the exception of calculating genetic indices, and to not influence direct comparisons due to exceptionally high sample size of Boa Vista, we randomly picked 100 sequences from Boa Vista and kept them for all downstream analyses. Results were corrected for multiple comparisons using the modified Benjamini-Yekutieli false discovery rate (FDR) method^67^, as suggested by Narum^68^. Furthermore, we calculated the average F_ST_ for each island-specific nesting group in order to investigate whether a geographic pattern of population structure exists across the archipelago.

#### Demographic history and colonization scenarios within the Cabo Verde archipelago

The demographic history of each island-specific nesting group was first investigated through moment estimates Tajima’s D (computed with 1000 coalescent simulations), sum of squares deviation (SSD), a measurement of goodness-of-fit, the raggedness index *r* and Fu´s Fs^69^. All these analyses were performed in Arlequin v3.5.1.3 and DNAsp v5.10^70^. Then, Bayesian skyline plots were constructed to infer fluctuations of effective population size throughout a temporal scale for each nesting group. These were computed in BEAST v.1.8^48^. The parameters substitution model and mutation rate were the same as the ones used in the phylogenetic scenarios. The initial tree root height and tree priors were also estimated in these analyses to have another perspective over colonization time for each island. Convergence was inspected in Tracer v1.6. Graphical display of the skylines was constructed in Tracer v1.6.

In order to further investigate the migration along the archipelago’s West–East axis, we calculated the effective number of migrants (ENI) per generation across islands and related it to geographic distance and direction with a linear model. Migration estimates were obtained with migrate-n. For this model, prior distribution for *gene flow* (M) and *effective population size* (θ) were set as uniform with upper and lower boundaries of θ = 0–100 and M = 0–1000. A total of 10^6^ steps were recorded in each chain after a burn-in of 10^4^ states. Two independent replicates were performed for each scenario within each run. A total of 2 × 10^8^ parameters values were visited. We ran it three times, averaged θ and M, and calculated effective migration rates (ENI) with the equation (θ_average_*M_average_)/2. Geographic distances were calculated from the GPS coordinates of each island. Direction was inferred in relation to the longitudinal position of each island pair. ENIs and geographic distances were log transformed and incorporated in a linear model as response and explanatory variables, respectively, while direction (eastwards or westwards) of gene flow was incorporated as a factor. Statistical analyses were conducted in R v3.2.3^71^.

#### Genetic diversity and structure within the island of Boa Vista

Fine-scale variation in the distribution of genetic diversity was investigated for turtles nesting on the island of Boa Vista across 7 different beaches. Boa Vista is the eastern most island of the Archipelago and has an area of 631.1 km^2^. It is the Cabo Verdean Island where the majority of the nesting events takes place^42^. Diversity indices (haplotype and nucleotide diversities), pairwise F_ST_ comparisons and exact tests of population differentiation amongst beaches were computed in Arlequin v3.5.1.3. Bayesian skylines were performed as mentioned above. Test were corrected for multiple testing using FDR.

## Supplementary information

Supplementary file1

Supplementary file2

Supplementary file3

Supplementary file4

Supplementary file5

Supplementary file6

Supplementary file7

Supplementary file8

Supplementary file9

Supplementary file10

Supplementary file11

Supplementary file12

## Data Availability

All sequences obtained are readily available on https://www.qmul.ac.uk/eizaguirrelab/turtlebase/ as part of the Turtle Project, a citizen-science program.
