# Supplementary figures and images for: Distribution of genetic diversity reveals colonization patterns and philopatry of the loggerhead sea turtles across geographic scales
Source: Sci Rep. 2020 Oct 22;10:18001. doi: 10.1038/s41598-020-74141-6 (PMC7583243; doi:10.1038/s41598-020-74141-6)

# Global and Global with frequency data

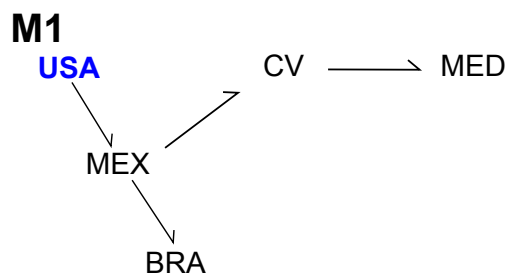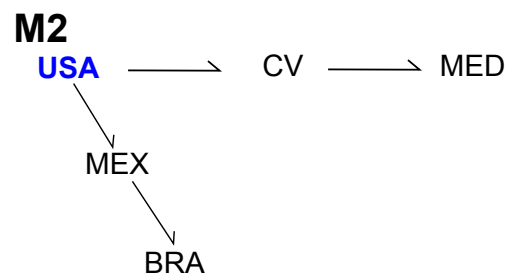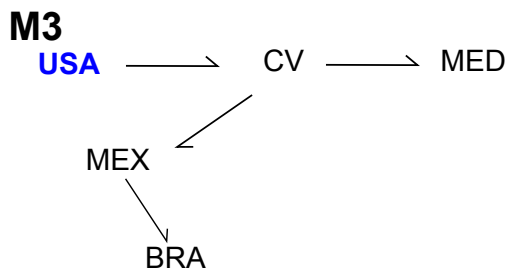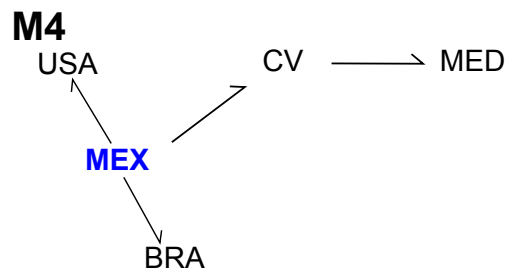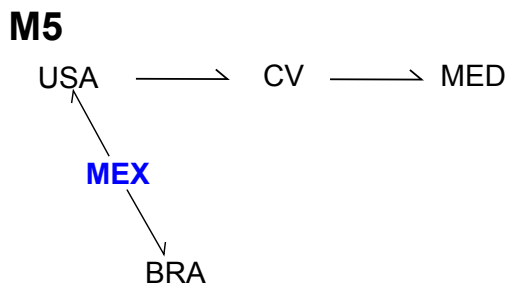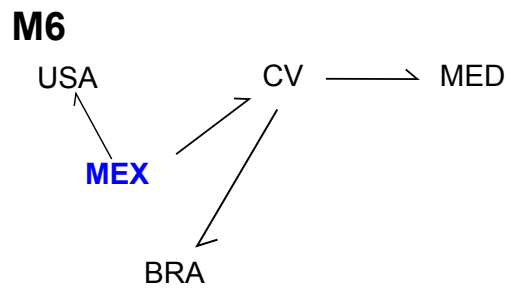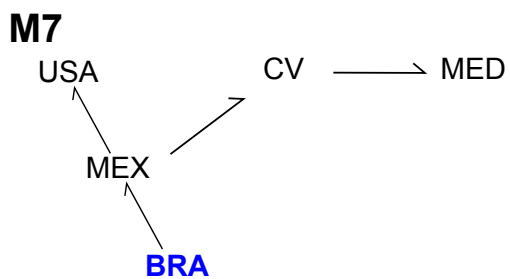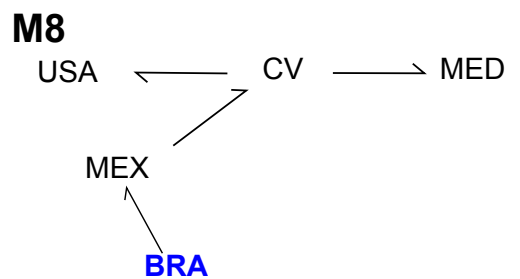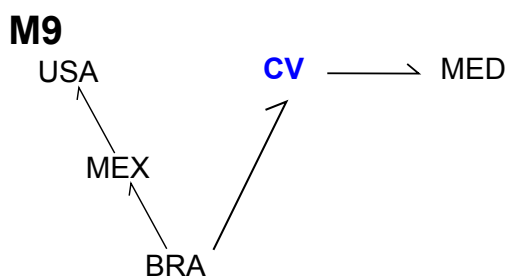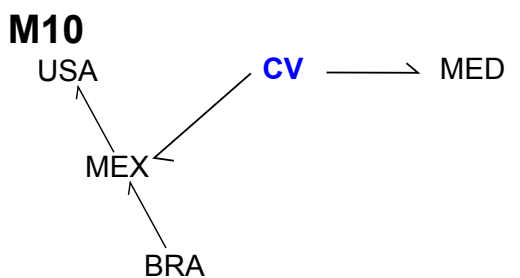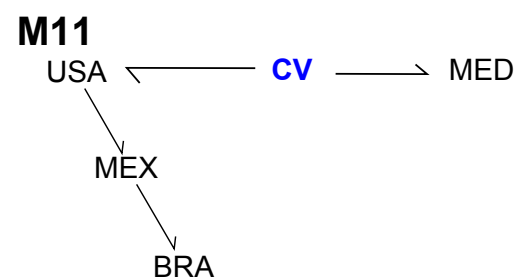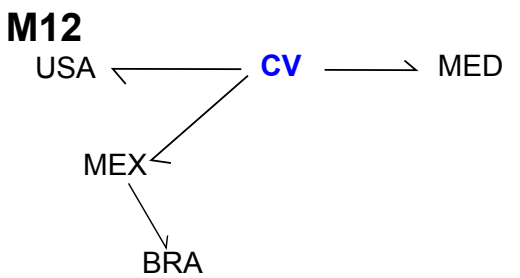

Supplement: Supplementary file 1 — Supplementary file1 [file 41598_2020_74141_MOESM1_ESM.pdf]

(a)

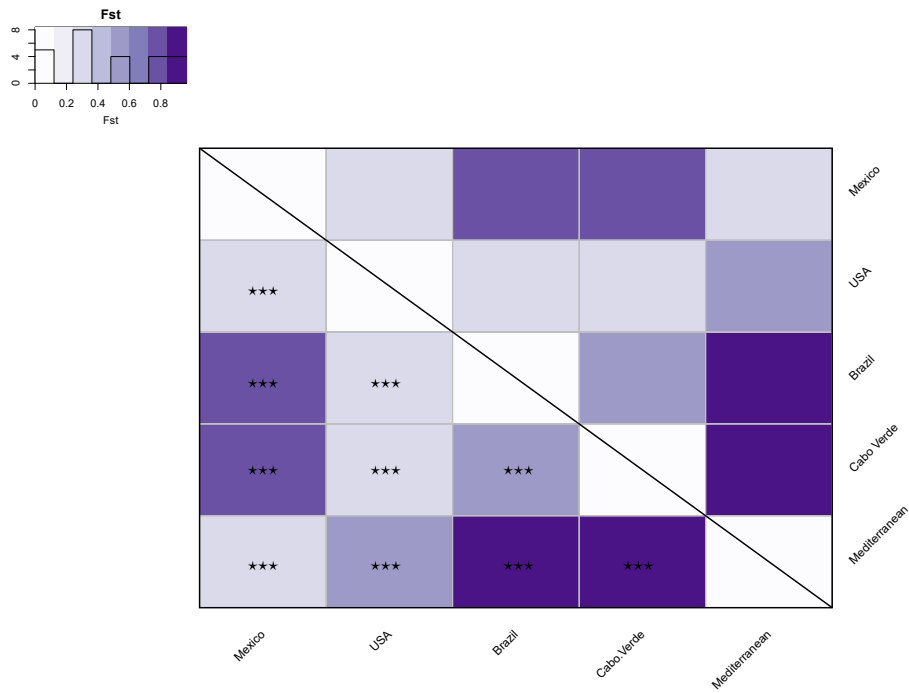

(b)

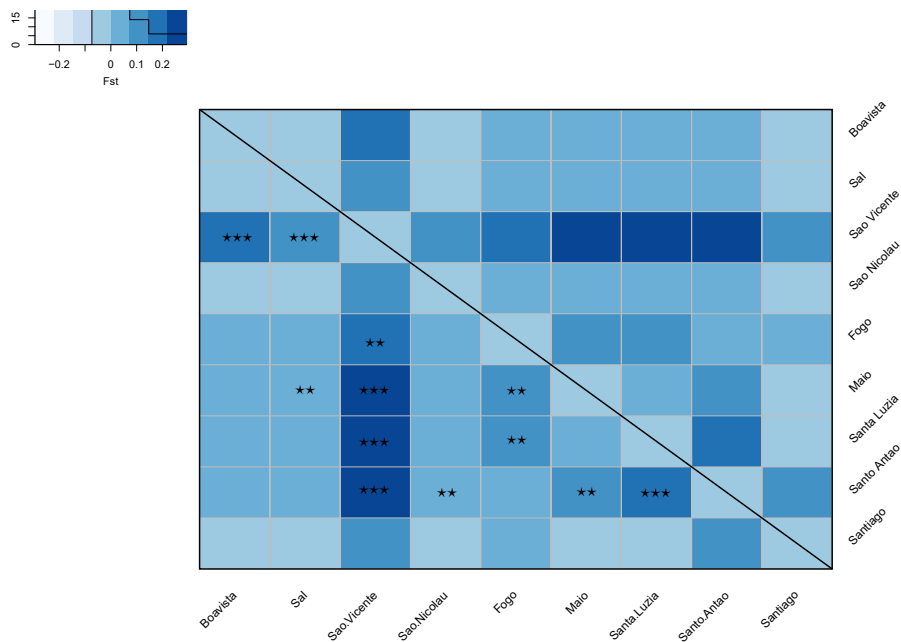

(c)

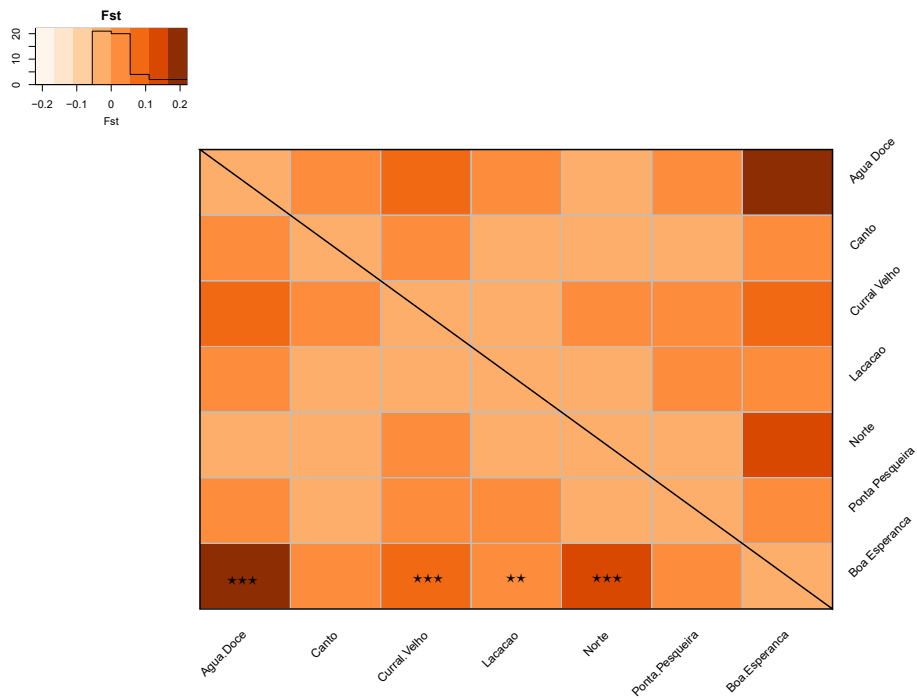

Supplement: Supplementary file 2 — Supplementary file2 [file 41598_2020_74141_MOESM2_ESM.pdf]

# Global

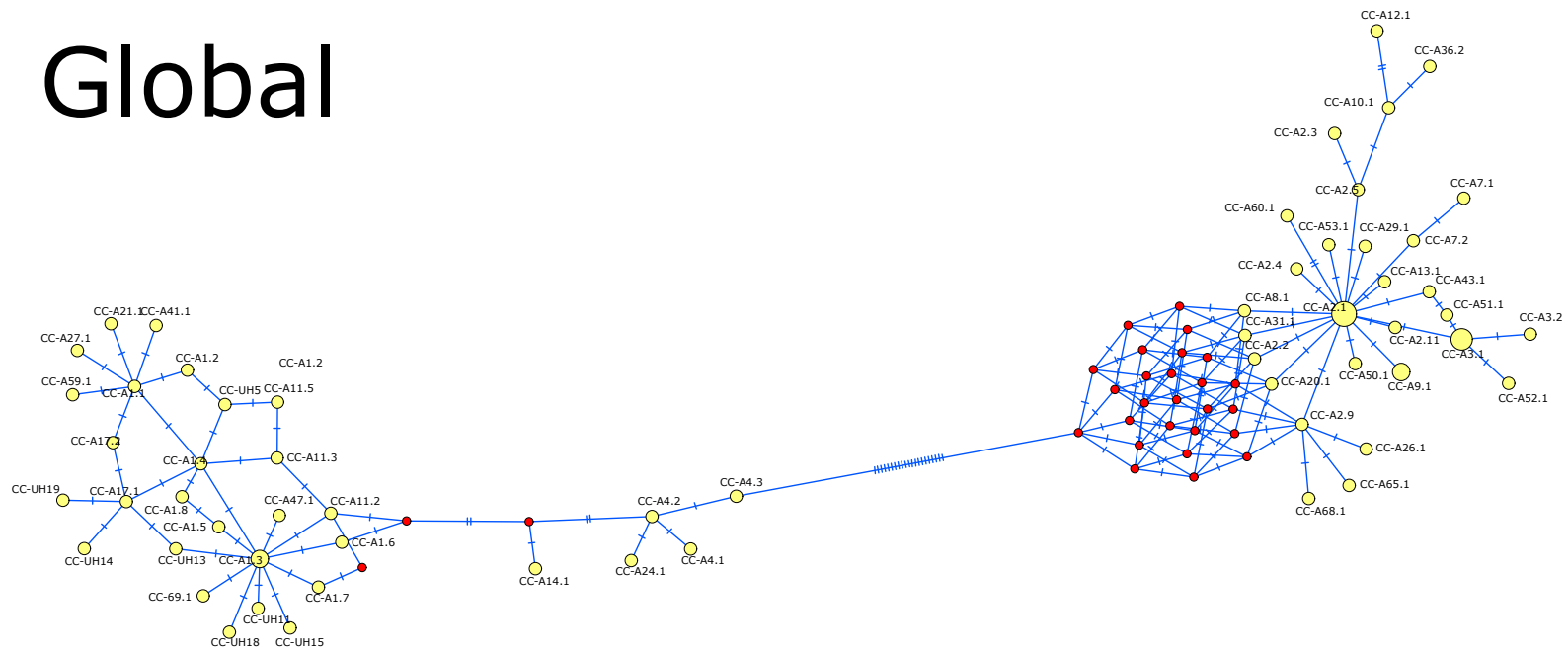

# Regional

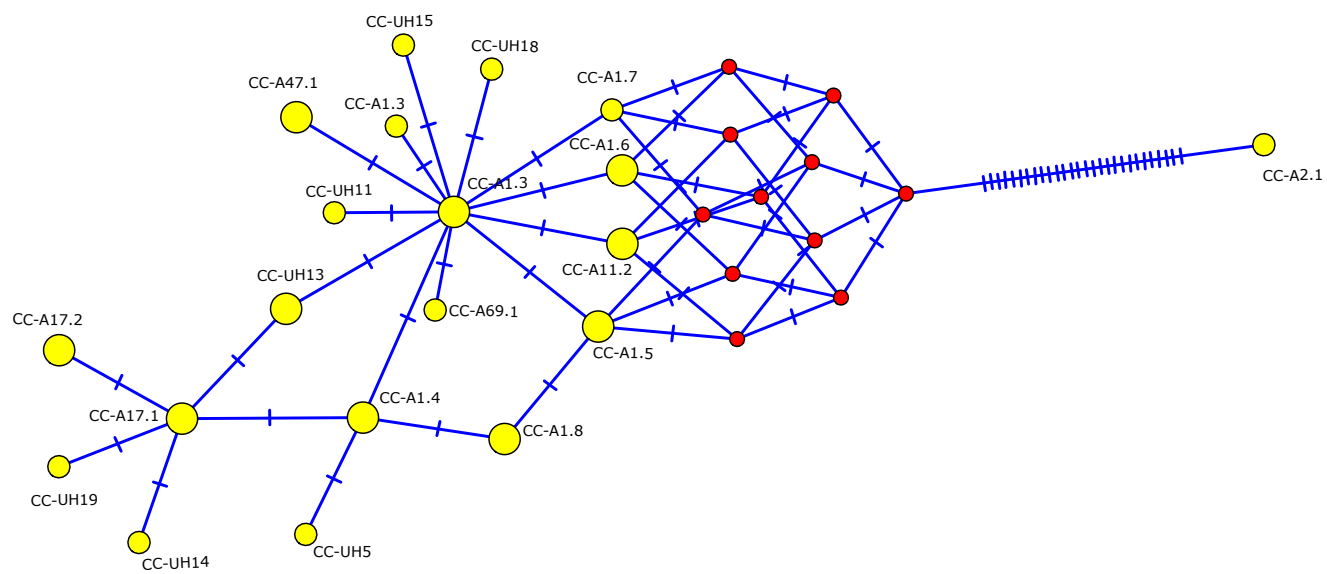

# Local

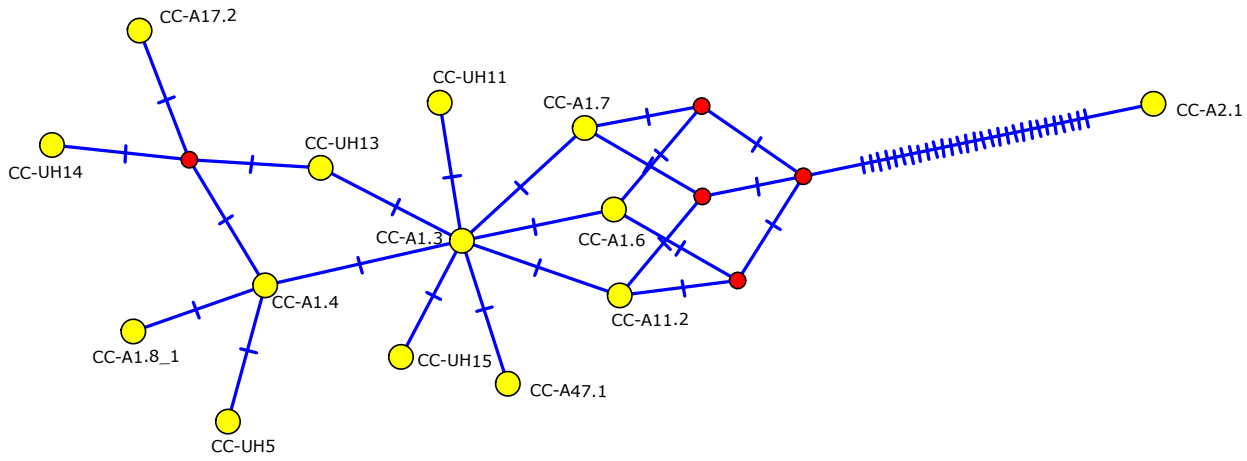

Supplement: Supplementary file 3 — Supplementary file3 [file 41598_2020_74141_MOESM3_ESM.pdf]

# Scenario I

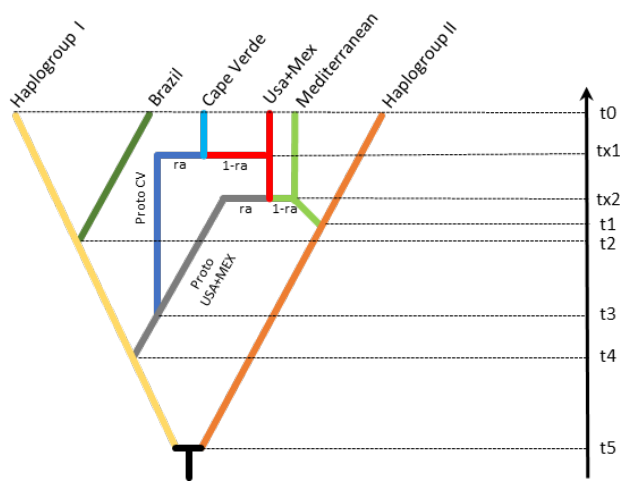

# Scenario II

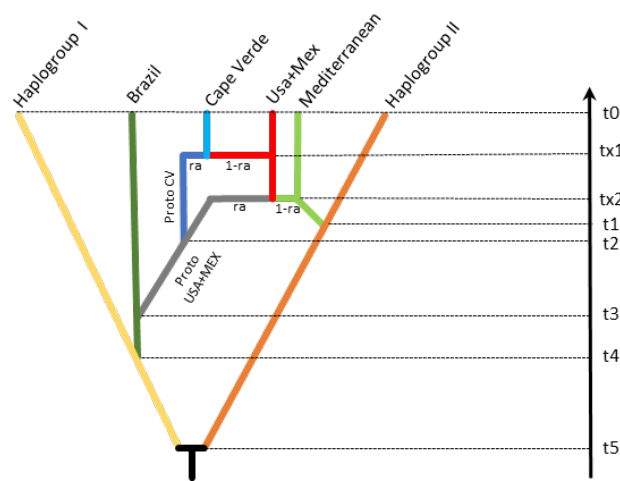

# Scenario III

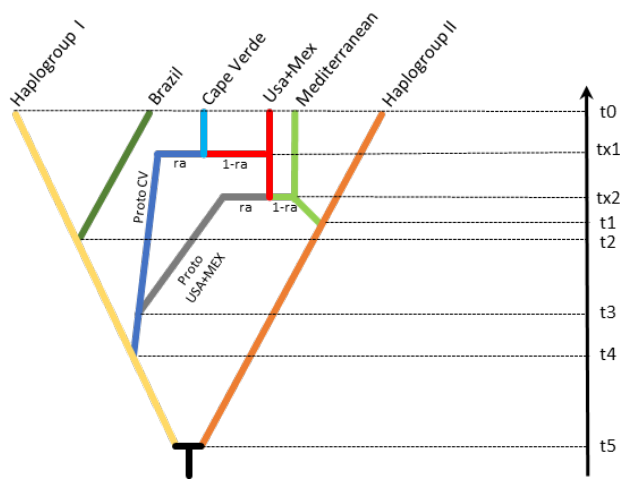

# Scenario IV

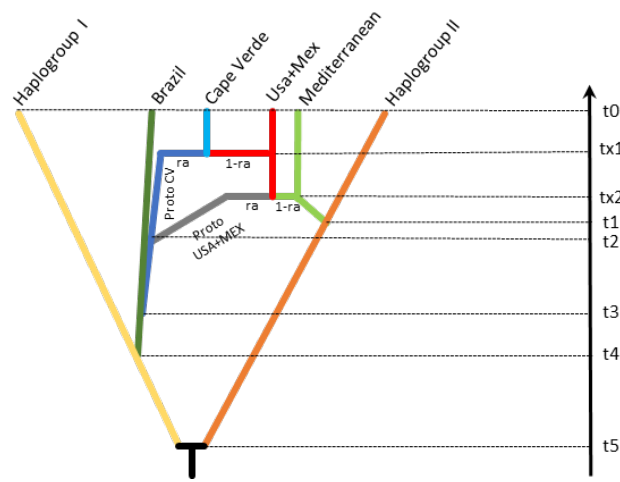

# Scenario V★

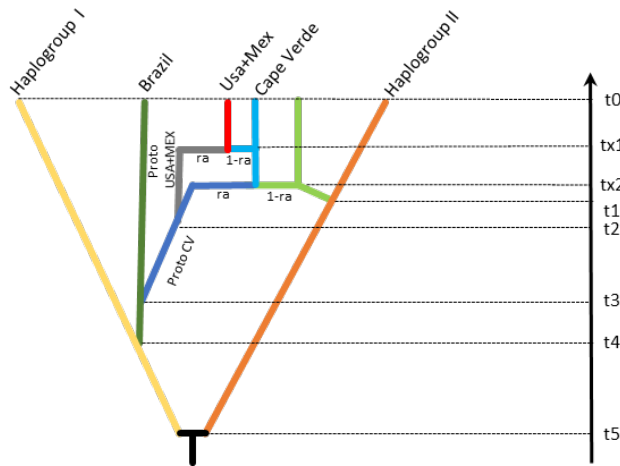

Supplement: Supplementary file 5 — Supplementary file5 [file 41598_2020_74141_MOESM5_ESM.pdf]

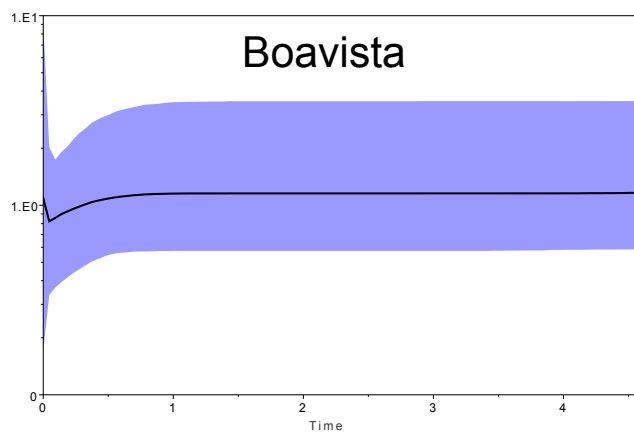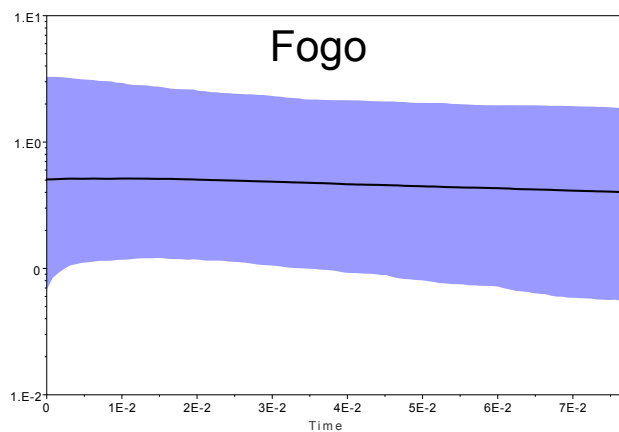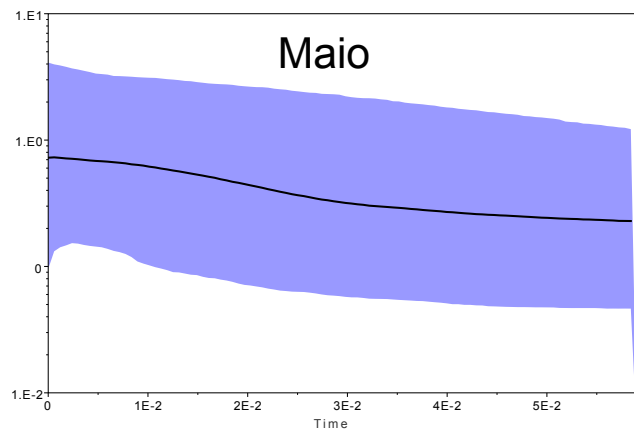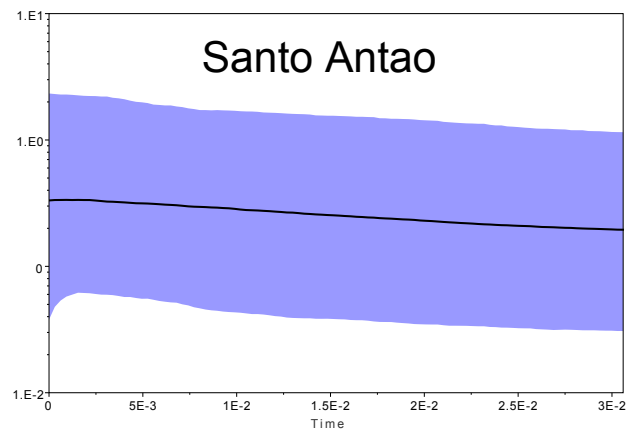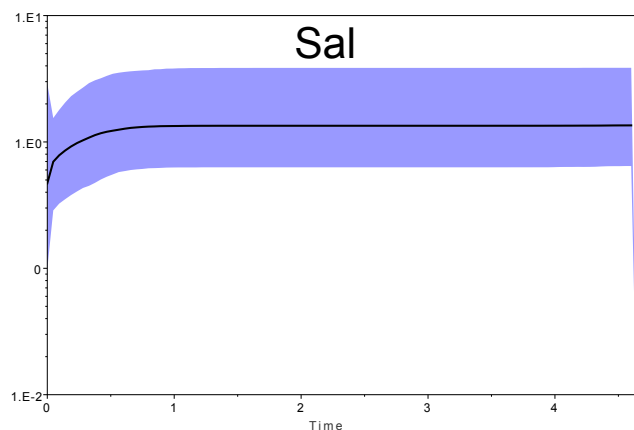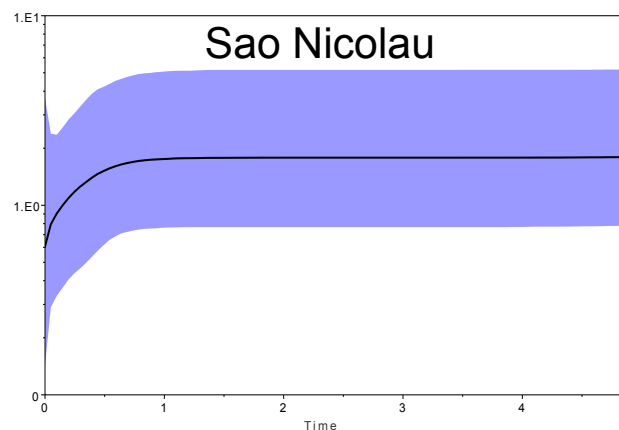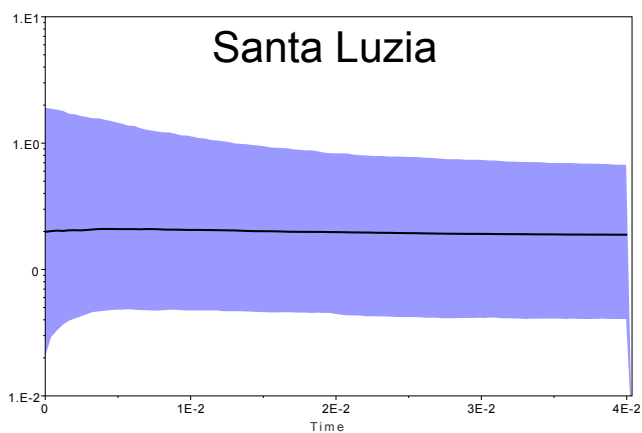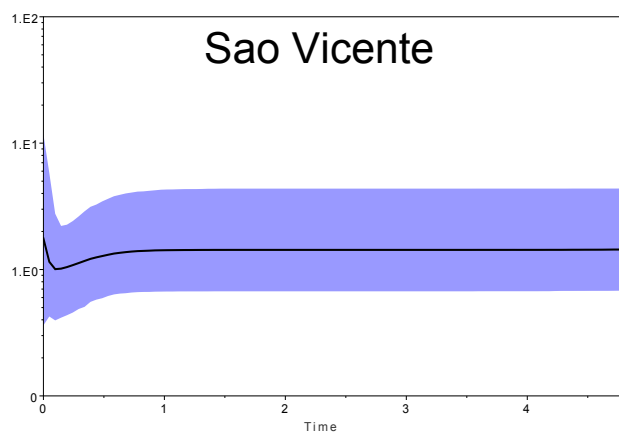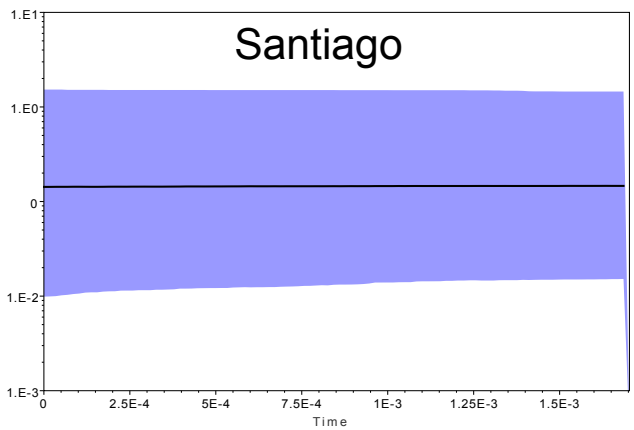

Supplement: Supplementary file 6 — Supplementary file6 [file 41598_2020_74141_MOESM6_ESM.pdf]

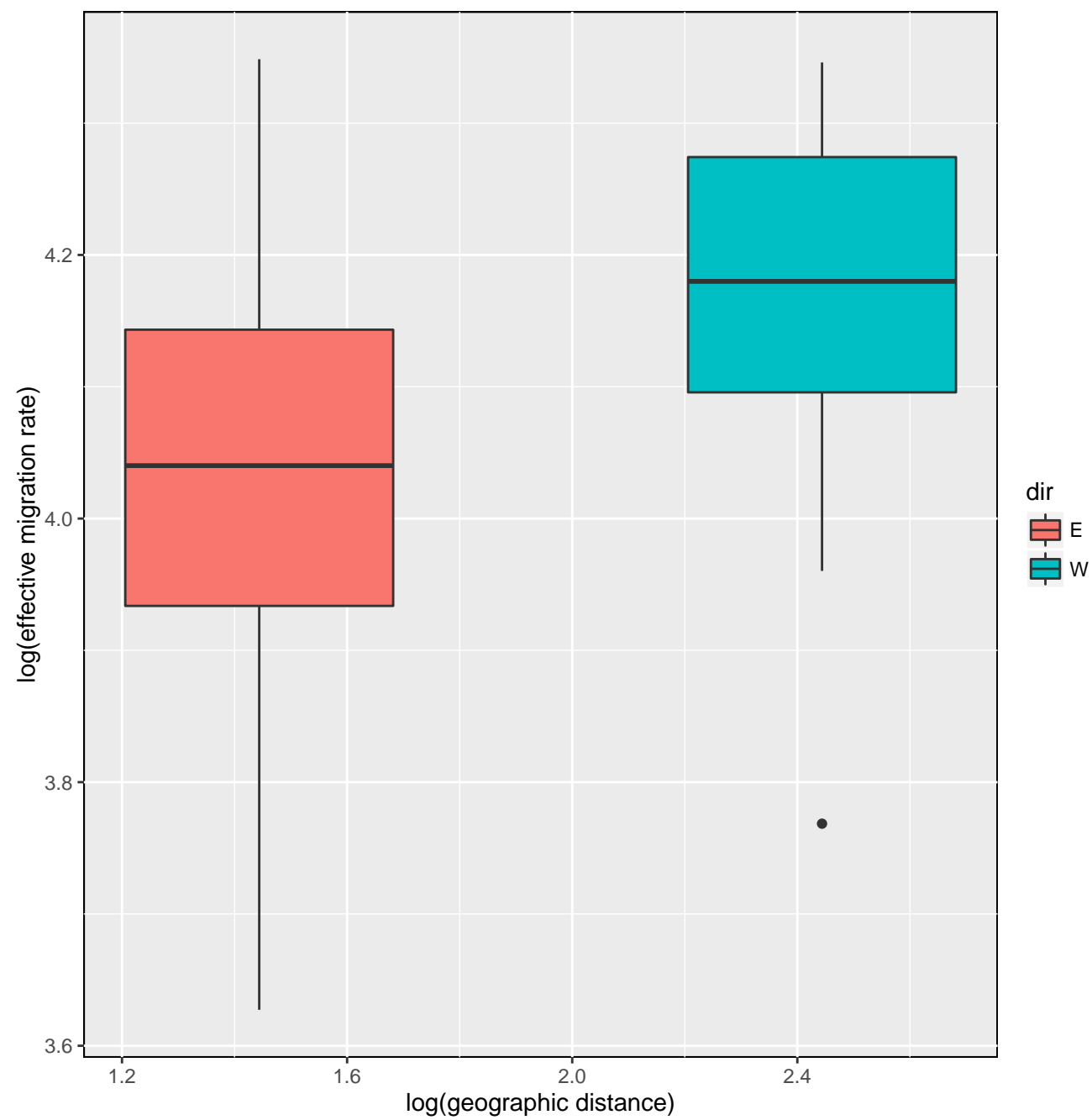

Supplement: Supplementary file 7 — Supplementary file7 [file 41598_2020_74141_MOESM7_ESM.pdf]

Agua Doce

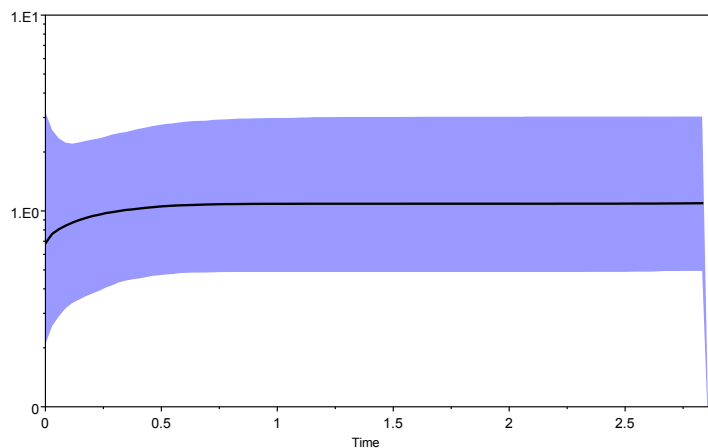

Boa Esperanca

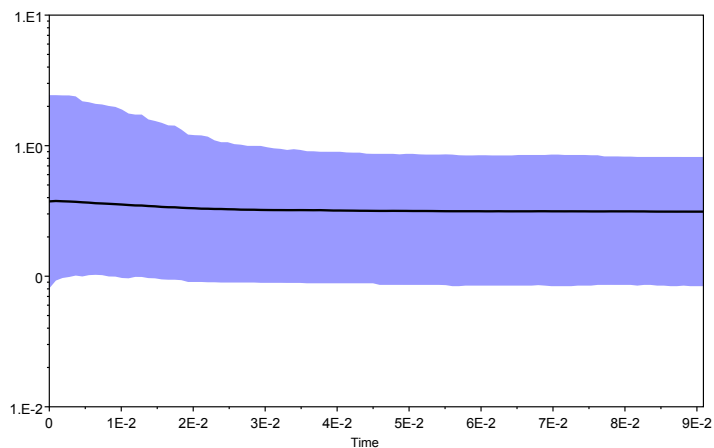

Canto

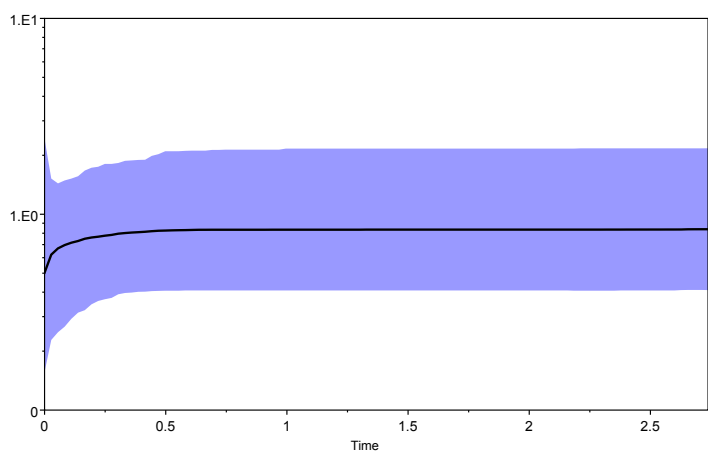

Curral Velho

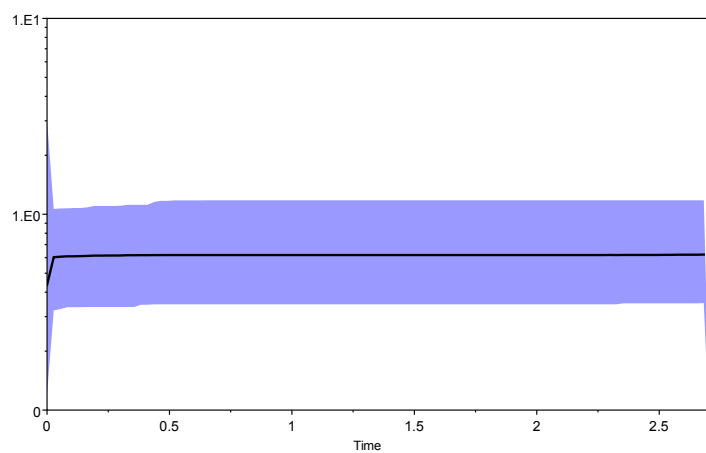

Norte

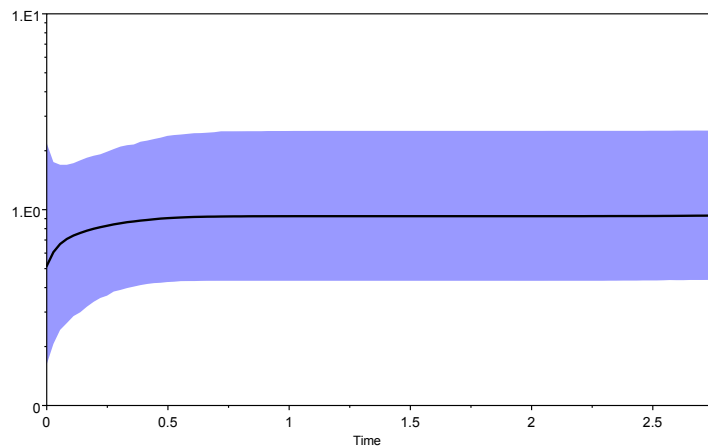

Ponta Pesqueira

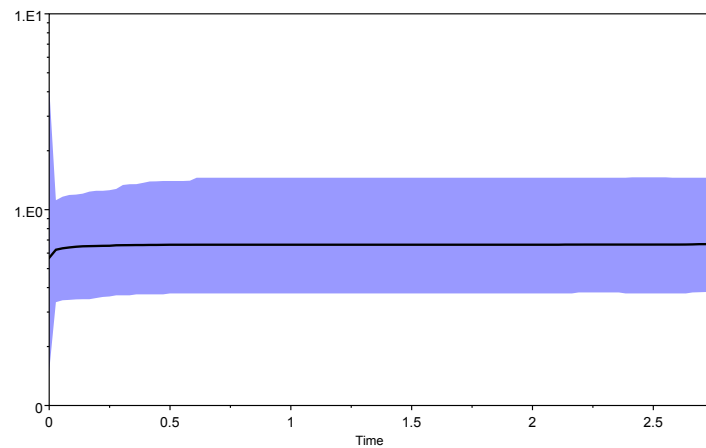

Lacacao

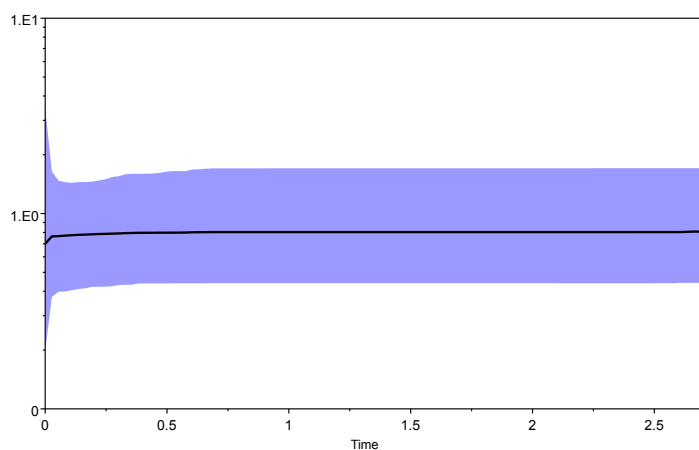

Supplement: Supplementary file 8 — Supplementary file8 [file 41598_2020_74141_MOESM8_ESM.pdf]

## Scenario I

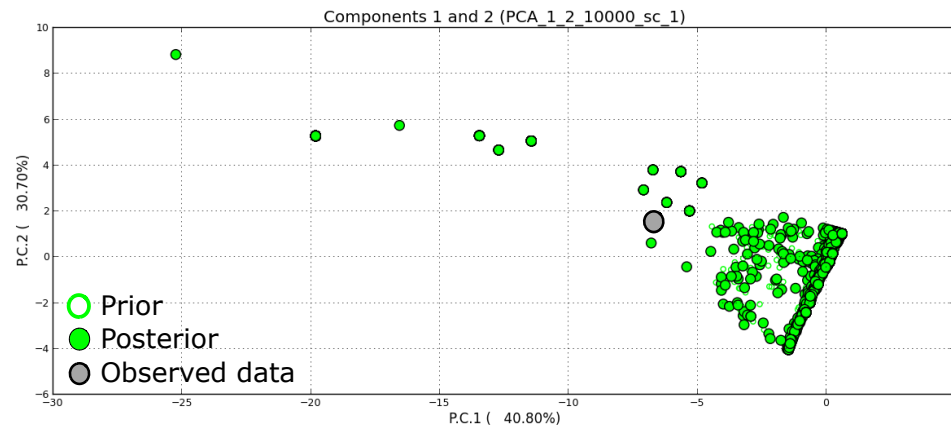

## Scenario II

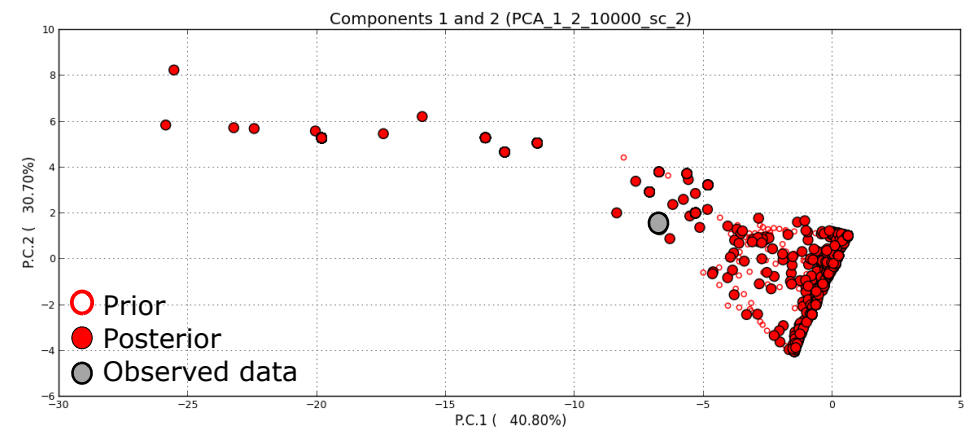

## Scenario III

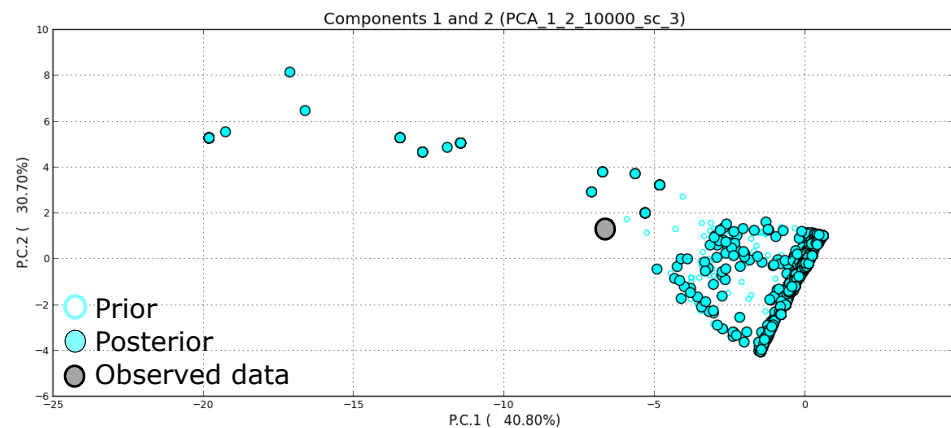

## Scenario IV

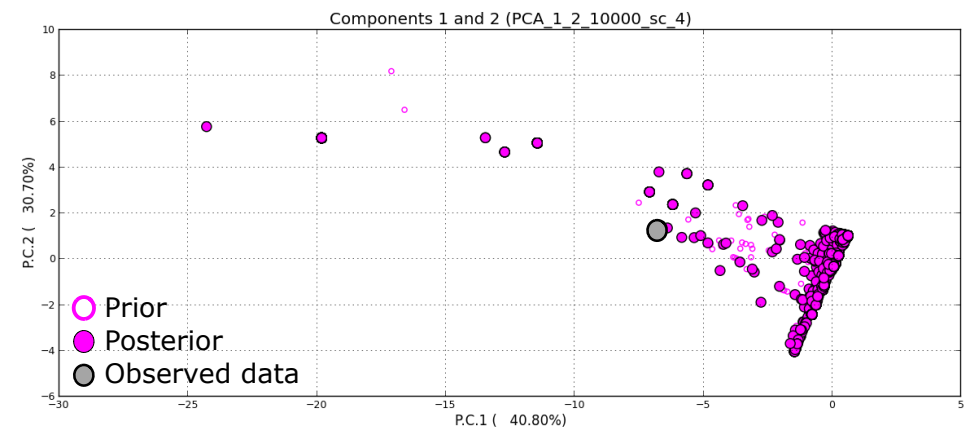

## Scenario V

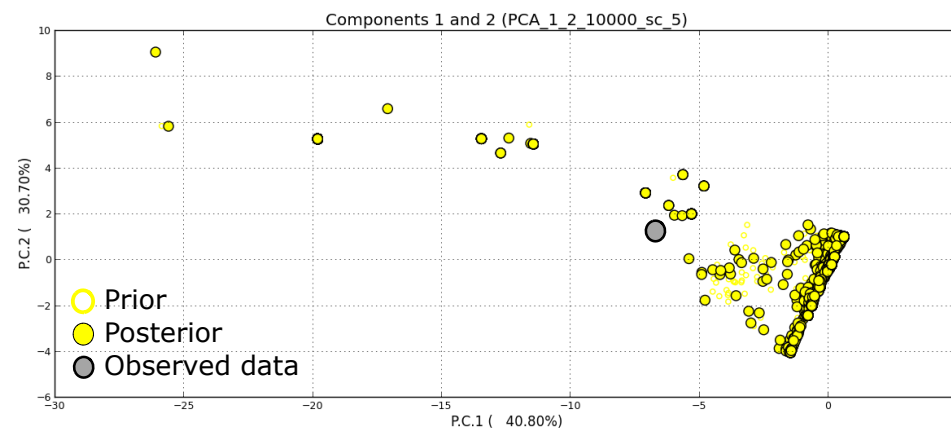

Supplement: Supplementary file 9 — Supplementary file9 [file 41598_2020_74141_MOESM9_ESM.pdf]
