# Supplementary material for: Distribution of genetic diversity reveals colonization patterns and philopatry of the loggerhead sea turtles across geographic scales
Source: Sci Rep. 2020 Oct 22;10:18001. doi: 10.1038/s41598-020-74141-6 (PMC7583243; doi:10.1038/s41598-020-74141-6)
Supplement: Supplementary file 12 — Supplementary file12 [file 41598_2020_74141_MOESM12_ESM.docx]

**Distribution of genetic diversity reveals colonization and philopatry of the loggerhead sea turtles across geographic scales**

Baltazar-Soares Miguel^1,2*^, Klein Juliana L.^3*^, Correia Sandra M.^4^, Reischig Thomas^5^, Taxonera Amoros Albert^6^, Monteiro Roque Silvana^7^, Dos Passos Leno^8^, Durão Jandira^9^, Pina Lomba João^10^, Dinis Herculano^11^, Cameron Sahmorie J.K.^1^, Stiebens Victor A.^1^, Eizaguirre Christophe ^1,#^

Affiliations:

^1^ School of Biological and Chemical Sciences, Queen Mary University of London, London, E1 4NS, UK

^2^ MARE-ISPA, Rua Jardim do Tabaco, 34, 1100-304 Lisboa Portugal

^3^ Centre for Ecological Genomics and Wildlife Conservation, Department of Zoology University of Johannesburg, Auckland Park, South Africa

^4^ Instituto do Mar (iMAr), Cova de Inglesa, C.P: 132 Mindelo, Sao Vicente, Cape Verde

^5^ Turtle Foundation, An der Eiche 7a, 50678 Cologne, Germany

^6^ Associação Projeto Biodiversidade, Mercado Municipal 22, Santa Maria 4111, Ilha do Sal, Cabo Verde

^7^ Projeto Vitó Porto Novo. Porto Novo, Santo Antão Island, Cabo Verde.

^8^ Foundation Maio Biodiversity, Cidade de Porto Inglês, Maio Island, Cabo Verde.

^9^ Biosfera I, Rua de Moçambique 28, Mindelo, São Vicente, Cabo Verde

^10^ Associação Ambiental Caretta Caretta. Achada Igreja, Pedra Badejo, Santa Cruz, Santiago, Cabo Verde^.^

^11^ Associação Projecto Vitó, Xaguate, São Felipe, Fogo, Cabo Verde.

* These authors contributed equally to this study

^#^ To whom correspondence should be addressed: [c.eizaguirre@qmul.ac.uk](mailto:c.eizaguirre@qmul.ac.uk)

**Table S1 - Pairwise F_ST_ among largest rookeries in the Atlantic.** Pairwise F_ST_ values are depicted in the lower diagonal. P values are shown in the upper diagonal. Significant results are shown in bold.

| ***Atlantic rookeries*** | | | | | |
| --- | --- | --- | --- | --- | --- |
|  | Mexico | USA | Brazil | Cape Verde | Mediterranean |
| Mexico | - | **0.000** | **0.000** | **0.000** | **0.000** |
| USA | **0.320** | - | **0.000** | **0.000** | **0.000** |
| Brazil | **0.782** | **0.309** | - | **0.000** | **0.000** |
| Cape Verde | **0.843** | **0.303** | **0.600** | - | **0.000** |
| Mediterranean | **0.261** | **0.495** | **0.964** | **0.927** | - |

**Table S2 – Haplotype frequencies among major rookeries.**

| ***Haplotype*** | ***USA*** | ***BRA*** | ***MED*** | ***MEX*** | ***CV*** |
| --- | --- | --- | --- | --- | --- |
| A1.1 | 0.541528 | 0 | 0 | 0 | 0 |
| A1.2 | 0.011391 | 0 | 0 | 0 | 0 |
| A1.3 | 0.010441 | 0 | 0 | 0.005714 | 0.604085 |
| A1.4 | 0.017561 | 0 | 0 | 0.074286 | 0.038492 |
| A1.5 | 0 | 0 | 0 | 0 | 0.00707 |
| A1.6 | 0 | 0 | 0 | 0 | 0.001571 |
| A1.7 | 0 | 0 | 0 | 0 | 0.00707 |
| A1.8 | 0 | 0 | 0 | 0 | 0.003142 |
| A1.9 | 0 | 0 | 0 | 0 | 0.003928 |
| A10.1 | 0.010916 | 0 | 0 | 0.234286 | 0 |
| A11.2 | 0 | 0 | 0 | 0 | 0.019639 |
| A11.3 | 0.000949 | 0 | 0 | 0.04 | 0 |
| A11.5 | 0 | 0 | 0 | 0.005714 | 0 |
| A11.6 | 0 | 0 | 0 | 0 | 0.005499 |
| A12.1 | 0 | 0 | 0 | 0.011429 | 0 |
| A13.1 | 0.001898 | 0 | 0.001919 | 0.011429 | 0 |
| A14.1 | 0.010441 | 0 | 0 | 0.04 | 0 |
| A17.1 | 0 | 0 | 0 | 0 | 0.232522 |
| A17.2 | 0 | 0 | 0 | 0 | 0.029851 |
| A2.1 | 0.306122 | 0 | 0.783109 | 0.365714 | 0.031422 |
| A2.11 | 0.000475 | 0 | 0 | 0 | 0 |
| A2.2 | 0.000475 | 0 | 0 | 0 | 0 |
| A2.3 | 0.002848 | 0 | 0 | 0.034286 | 0 |
| A2.4 | 0.005695 | 0 | 0 | 0 | 0 |
| A2.5 | 0.002373 | 0 | 0 | 0.057143 | 0 |
| A2.8 | 0 | 0 | 0.007678 | 0 | 0 |
| A2.9 | 0 | 0 | 0.028791 | 0 | 0 |
| A20.1 | 0.004746 | 0 | 0.026871 | 0 | 0 |
| A21.1 | 0.000949 | 0 | 0 | 0 | 0 |
| A24.1 | 0 | 0.038168 | 0 | 0 | 0 |
| A26.1 | 0 | 0 | 0.007678 | 0 | 0 |
| A27.1 | 0.000475 | 0 | 0 | 0 | 0 |
| A29.1 | 0 | 0 | 0.003839 | 0 | 0 |
| A3.1 | 0.053156 | 0 | 0.111324 | 0.017143 | 0 |
| A3.2 | 0 | 0 | 0.001919 | 0 | 0 |
| A31.1 | 0 | 0 | 0.005758 | 0 | 0 |
| A36.1 | 0.000475 | 0 | 0 | 0 | 0 |
| A36.2 | 0.000475 | 0 | 0 | 0.005714 | 0 |
| A4.1 | 0 | 0.282443 | 0 | 0 | 0 |
| A4.2 | 0 | 0.648855 | 0 | 0 | 0 |
| A4.3 | 0 | 0.030534 | 0 | 0 | 0 |
| A41.1 | 0.000475 | 0 | 0 | 0 | 0 |
| A42.1 | 0 | 0 | 0 | 0.005714 | 0 |
| A43.1 | 0.000475 | 0 | 0 | 0 | 0 |
| A47.1 | 0 | 0 | 0 | 0 | 0.004713 |
| A5.1 | 0.000949 | 0 | 0 | 0.011429 | 0 |
| A50.1 | 0 | 0 | 0.001919 | 0 | 0 |
| A51.1 | 0.000949 | 0 | 0 | 0 | 0 |
| A52.1 | 0 | 0 | 0.001919 | 0 | 0 |
| A53.1 | 0 | 0 | 0.003839 | 0 | 0 |
| A59.1 | 0.000475 | 0 | 0 | 0 | 0 |
| A6.1 | 0 | 0 | 0.009597 | 0 | 0 |
| A60.1 | 0 | 0 | 0 | 0.005714 | 0 |
| A68.1 | 0 | 0 | 0.003839 | 0 | 0 |
| A69.1 | 0 | 0 | 0 | 0 | 0.000786 |
| A7.1 | 0.007119 | 0 | 0 | 0 | 0 |
| A7.2 | 0.000949 | 0 | 0 | 0 | 0 |
| A8.1 | 0.000949 | 0 | 0 | 0.034286 | 0 |
| A9.1 | 0.004271 | 0 | 0 | 0.04 | 0 |
| UH5 | 0 | 0 | 0 | 0 | 0.003142 |
| UH11 | 0 | 0 | 0 | 0 | 0.000786 |
| UH13 | 0 | 0 | 0 | 0 | 0.001571 |
| UH14 | 0 | 0 | 0 | 0 | 0.000786 |
| UH15 | 0 | 0 | 0 | 0 | 0.000786 |
| UH17 | 0 | 0 | 0 | 0 | 0.000786 |
| UH18 | 0 | 0 | 0 | 0 | 0.000786 |
| UH19 | 0 | 0 | 0 | 0 | 0.001571 |

**Table S3 – Comparison of ancestrality models performed with BEAST.** Shown in bold is the best fit model for the ancestral rookery.

|  | AICM | SE | Brazil | Cape Verde | Mediterranean | Mexico | USA |
| --- | --- | --- | --- | --- | --- | --- | --- |
| Brazil | 11531.545 | +/- 1.399 | - | -47.275 | -22.133 | -51.503 | -68.486 |
| Cape Verde | 11484.27 | +/- 2.21 | 47.275 | - | 25.142 | -4.228 | -21.211 |
| Mediterranean | 11509.413 | +/- 1.328 | 22.133 | -25.142 | - | -29.37 | -46.353 |
| Mexico | 11480.043 | +/- 1.119 | 51.503 | 4.228 | 29.37 | - | -16.983 |
| **USA** | **11463.059** | **+/- 0.97** | **68.486** | **21.211** | **46.353** | **16.983** | **-** |

**Table S4 – Comparison of the Atlantic colonization scenarios computed in migrate-n.** Highlighted in green are the top 3 ranked models

| **Global** | | | | |
| --- | --- | --- | --- | --- |
|  | ML | LBF | probability | rank |
| M1 | -2219.01 | -16.129 | 0.000 | 0 |
| M2 | -2203.67 | -0.787 | 0.287 | 2 |
| M3 | -2215.75 | -12.869 | 0.000 | 0 |
| M4 | -2202.88 | 0.000 | 0.631 | 1 |
| M5 | -2216.65 | -13.765 | 0.000 | 0 |
| M6 | -2208.74 | -5.853 | 0.002 | 0 |
| M7 | -2214.7 | -11.816 | 0.000 | 0 |
| M8 | -2222.7 | -19.823 | 0.000 | 0 |
| M9 | -2229.24 | -26.360 | 0.000 | 0 |
| M10 | -2214.68 | -11.794 | 0.000 | 0 |
| M11 | -2204.95 | -2.066 | 0.080 | 3 |
| M12 | -2235.5 | -32.620 | 0.000 | 0 |

**Table S5 - Pairwise F_ST_ among islands of Cabo Verde.** Pairwise F_ST_ values are depicted in the lower diagonal. P values are shown in the upper diagonal. Significant results are shown in bold.

| ***Cabo Verde islands*** | | | | | | | | | |
| --- | --- | --- | --- | --- | --- | --- | --- | --- | --- |
|  | Boavista | Sal | Sao Vicente | Sao Nicolau | Fogo | Maio | Santa Luzia | Santo Antao | Santiago |
| Boavista | - | 0.453 | **0.000** | 0.763 | 0.071 | 0.074 | 0.112 | 0.018 | 0.983 |
| Sal | -0.004 | - | **0.000** | 0.841 | 0.121 | **0.002** | 0.028 | 0.025 | 0.457 |
| Sao Vicente | **0.188** | **0.144** | - | 0.027 | **0.005** | **0.000** | **0.000** | **0.000** | 0.016 |
| Sao Nicolau | -0.021 | 0.021 | 0.114 | - | 0.036 | 0.075 | 0.106 | **0.002** | 0.735 |
| Fogo | 0.024 | 0.021 | **0.174** | 0.031 | - | **0.006** | **0.003** | 0.024 | 0.108 |
| Maio | 0.017 | **0.039** | **0.294** | 0.037 | **0.097** | - | 0.282 | **0.000** | 0.930 |
| Santa Luzia | 0.014 | 0.033 | **0.235** | 0.025 | **0.143** | 0.002 | - | **0.000** | 0.635 |
| Santo Antao | 0.043 | 0.044 | **0.238** | **0.073** | 0.057 | **0.128** | **0.193** | - | 0.027 |
| Santiago | -0.033 | 0.017 | 0.134 | -0.037 | 0.072 | 0.048 | -0.035 | 0.135 | - |

**Table S6 – Pairwise F_ST_ among beaches of Boavista.** Pairwise F_ST_ values are depicted in the lower diagonal. P values are shown in the upper diagonal. Significant results are shown in bold.

| ***Boavista beaches*** | | | | | | | |
| --- | --- | --- | --- | --- | --- | --- | --- |
|  | Agua Doce | Canto | Curral Velho | Lacacao | Norte | Ponta Pesqueira | Boa Esperanca |
| Agua Doce | - | 0.145 | 0.040 | 0.077 | 0.700 | 0.176 | **0.000** |
| Canto | 0.013 | - | 0.156 | 0.433 | 0.585 | 0.844 | 0.088 |
| Curral Velho | 0.059 | 0.008 | - | 0.815 | 0.275 | 0.020 | **0.000** |
| Lacacao | 0.037 | 0.005 | -0.006 | - | 0.350 | 0.207 | **0.002** |
| Norte | -0.047 | 0.018 | 0.006 | -0.003 | - | 0.671 | **0.000** |
| Ponta Pesqueira | 0.008 | 0.014 | 0.014 | 0.004 | 0.016 | - | 0.035 |
| Boa Esperanca | **0.220** | 0.018 | **0.062** | **0.051** | **0.114** | 0.026 | - |
