# Supplementary material for: Distribution of genetic diversity reveals colonization patterns and philopatry of the loggerhead sea turtles across geographic scales
Source: Sci Rep. 2020 Oct 22;10:18001. doi: 10.1038/s41598-020-74141-6 (PMC7583243; doi:10.1038/s41598-020-74141-6)
Supplement: Supplementary file 4 — Supplementary file4 [file 41598_2020_74141_MOESM4_ESM.pdf]

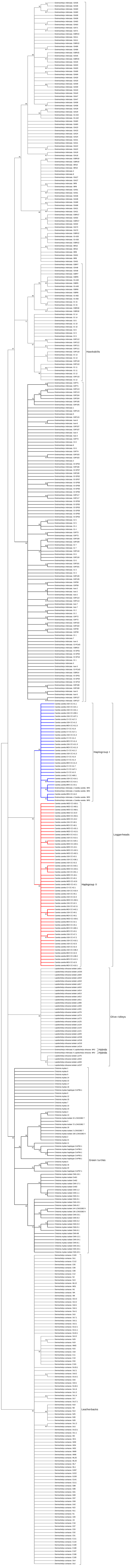
Hawksbills
Haplogroup I
Loggerheads
Haplogroup II
Hybrids
Hybrids
Olive ridleys
Green turtles
Leatherbacks
